# Mapping integral cell-type-specific interferon-induced gene regulatory networks (GRNs) involved in systemic lupus erythematosus using systems and computational analysis

**DOI:** 10.1016/j.heliyon.2024.e41342

**Published:** 2024-12-18

**Authors:** Blessy Kiruba, Akshayata Naidu, Vino Sundararajan, Sajitha Lulu S

**Affiliations:** a Department of Biosciences, School of Bio Sciences and Technology, Vellore Institute of Technology, Vellore, 632 014, Tamil Nadu, India; b Department of Biotechnology, School of Bio Sciences and Technology, Vellore Institute of Technology, Vellore, 632 014, Tamil Nadu, India; c Department of Biosciences, School of Bio Sciences and Technology, Vellore Institute of Technology, Vellore, 632 014, Tamil Nadu, India

**Keywords:** Systemic lupus erythematosus, Gene regulatory networks, Non-coding RNAs, Interferons, Targets

## Abstract

Systemic lupus erythematosus (SLE) is a systemic autoimmune disorder characterized by the production of autoantibodies, resulting in inflammation and organ damage. Although extensive research has been conducted on SLE pathogenesis, a comprehensive understanding of its molecular landscape in different cell types has not been achieved. This study uncovers the molecular mechanisms of the disease by thoroughly examining gene regulatory networks within neutrophils, dendritic cells, T cells, and B cells. Firstly, we identified genes and ncRNAs with differential expression in SLE patients compared to controls for different cell types. Furthermore, the derived differentially expressed genes were curated based on immune functions using functional enrichment analysis to create a protein-protein interaction network. Topological network analysis of the formed genes revealed key hub genes associated with each of the cell types. To understand the regulatory mechanism through which these hub genes function in the diseased state, their associations with transcription factors, and non-coding RNAs in different immune cell types were investigated through correlation analysis and regression models. Finally, by integrating these findings, distinct gene regulatory networks were constructed, which provide a novel perspective on the molecular, cellular, and immunological landscapes of SLE. Importantly, we reveal the crucial role of IRF3, IRF7, and STAT1 in neutrophils, dendritic cells, and T cells, where their aberrant upregulation in disease states might enhance the production of type I IFN. Furthermore, we found MYB to be a crucial regulator that might activate T cells toward autoimmune responses in SLE. Similarly, in B-cell lymphocytes, we found FOXO1 to be a key player in autophagy and chemokine regulation. These findings were also validated using single-cell RNASeq analysis using an independent dataset. Genotype variations of these genes were also explored using the GWAS central database to ensure their targetability. These findings indicate that IRF3, IRF7, STAT1, MYB, and FOXO1 are promising targets for therapeutic interventions for SLE.

## Highlights

• Distinct gene regulatory networks were constructed for neutrophils, dendritic cells, T cells, and B cells from SLE patients.
• IRF3, IRF7, and STAT1 play crucial roles in type I IFN production in neutrophils, dendritic cells, and T cells.
• MYB acts as a key regulator in T cell activation, promoting autoimmunity in SLE.
• FOXO1 triggers autophagy and chemokine regulation in B cells, influencing immune responses.

## Graphical abstract

**Figure:**
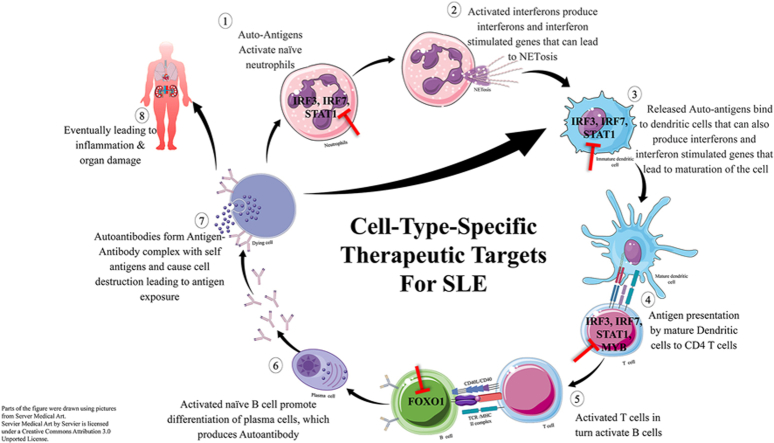

## Teaser

**SUMMARY** This study investigated the molecular dynamics of systemic lupus erythematosus (SLE) by examining gene regulatory networks in different immune cells. The findings reveal distinct regulatory networks in neutrophils, dendritic cells, T cells, and B cells, highlighting the roles of IRF3, IRF7, STAT1, MYB, and FOXO1 in disease progression. These findings suggest that these genes are potential therapeutic targets for SLE.

## 1 Introduction

Systemic lupus erythematosus (SLE) is a multifaceted autoimmune disorder marked by the generation of autoantibodies targeting self-antigens, resulting in widespread tissue inflammation and progressive organ damage. The pathogenesis of SLE is complex but two key immune features—loss of tolerance to nucleic acids and increased interferon production—play a central role in its development [1]. Research indicates that an interferon signature is closely associated with the severity of SLE manifestations [[2], [3], [4], [5]]. Additionally, various mechanisms drive disease progression, starting with impaired clearance of cellular debris leading to accumulation of nucleic acids, which can cause activation of toll-like receptors on antigen-presenting cells that trigger the interferon pathway. Type I IFN enhances the humoral immune response, promoting autoantibody production [6,7]. This cascade activates the innate immune system—primarily neutrophils, dendritic cells, macrophages, and monocytes—followed by an autoimmune response mediated by the adaptive immune system, including B cells, T cells, and plasma cells, which collectively intensifies the disease progression [8]. Though several genetic and epigenetic factors play a vital role in causing SLE, in recent studies non-coding RNAs (ncRNAs) appear to have a crucial contribution to the disease pathology [9,10]. Long non-coding RNAs (lncRNAs) and microRNAs (miRNAs) especially play crucial roles in immune system development and regulating adaptive and innate immune responses. Consequently, disruptions in their expression and function are pivotal in driving the onset and progression of autoimmune diseases, including SLE [11].

Despite significant progress in understanding and managing SLE, finding a definitive cure remains elusive [12]. There is also an urgent need for improved diagnostic tools, identification of therapeutic targets, and optimization of treatment options [13]. Moreover, understanding the cellular and molecular complexity of SLE is essential for developing personalized treatments and advancing precision medicine, which continue to present significant challenges in disease management [14]. Hence, we undertook this study to understand and explore the distinct gene regulatory networks associated with the molecular pathogenesis of SLE within different immune cell types. Specifically, the study aims to identify key regulatory genes and ncRNAs that contribute to disease progression and can serve as potential therapeutic targets.

To achieve our objectives, we implemented a comprehensive pipeline that combines computational and network biology approaches to analyze an extensive corpus of bulk RNA-seq data, used as a discovery cohort. This allowed us to profile gene expression across specific immune cell types—neutrophils, dendritic cells, T cells, and B cells—isolated via methods such as fluorescence-activated cell sorting (FACS). Through this approach, we identified differentially expressed genes, constructed protein-protein interaction networks, and developed gene regulatory networks specific to each cell type. Single-cell RNA sequencing was employed as a validation cohort to further validate our findings, refining and confirming the insights gained from bulk RNA-seq.

## 2 Materials and methods

### 2.1 RNA-seq data collection and processing

RNA sequencing (RNASeq) has emerged as a powerful tool in molecular biology, enabling researchers to comprehensively study gene expression patterns in diverse biological systems. This approach provides insights into the transcriptional landscape and can be used to directly measure gene expression levels, unlike genomic analysis which primarily reveals genetic variations. This also enables the identification of genes actively involved in cellular processes and disease mechanisms. Moreover, lincRNAs and miRNAs that play a significant role in gene regulation can also be identified.

**Fig. 1:**
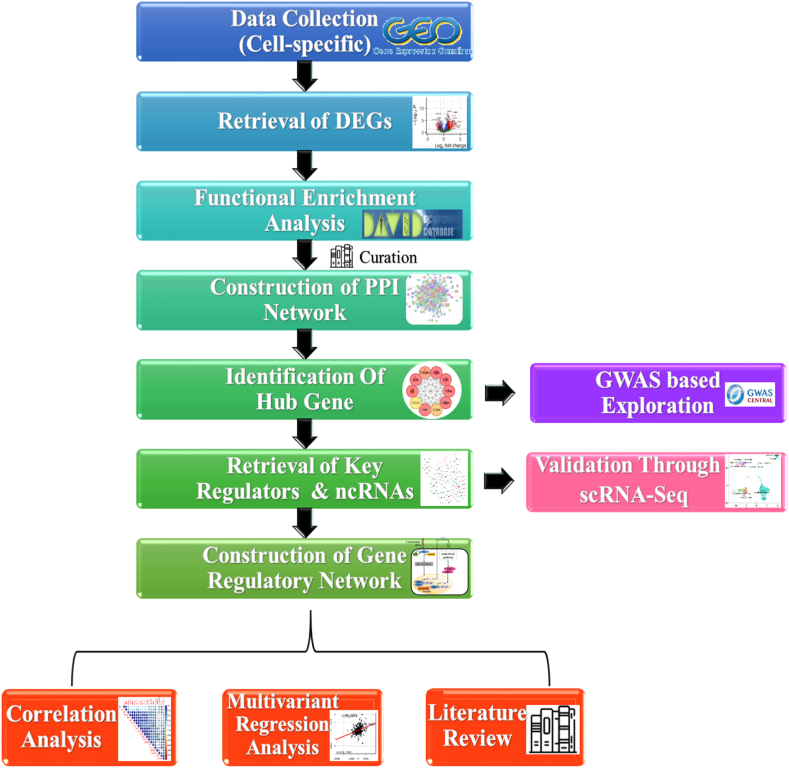
Schematic representation of the research workflow.

For our study, RNA-Seq data for SLE patients were retrieved from the Gene Expression Omnibus (GEO) database. Different cells/tissue samples namely, neutrophils, dendritic cells, T cells, and B cells, that had distinct samples of control and SLE were retrieved for further analysis. The neutrophil dataset included 6 new-onset treatment-naïve SLE patients and 6 age/sex-matched healthy controls, while from the dendritic cell dataset, 11 controls and 17 SLE samples were retrieved. Additionally, for T cell, 13 SLE active samples and age-matched and ethnicity-matched 14 controls were retrieved while the B cell dataset consisted of 37 controls and 48 SLE samples. The accession IDs of the different datasets selected are listed in Table 1. Furthermore, an overview of the workflow of our study is illustrated in Fig. 1.

**Table 1:** RNA-Seq data with the accession number for each cell/tissue.

GEO Accession ID	Cells/Tissue	No. of Samples	Location	Ref. Publ
GSE153781	Neutrophils	12	China	N/A
GSE136731	cDC2 dendritic cells	47	Singapore	722
GSE118254	B cells	83	USA	733
GSE97263	T cells	44	UK	744

### 2.2 Retrieval of differentially expressed genes

To identify differentially expressed genes (DEGs) and non-coding RNAs (ncRNAs) between diseased and normal samples across all four datasets, we used GEO2R, an analysis tool provided by GEO. GEO2R is an interactive tool designed for comparing multiple sample groups within a GEO Series, allowing users to identify genes that are differentially expressed. In brief, the workflow began by aligning reads to the human genome (GCA_000001405.15) using HISAT2. Only samples with an alignment rate above 50% were retained for further analysis. These aligned reads were then processed with Subread featureCounts to produce raw count files. Futhermore, for gene annotations, the Homo sapiens Annotation Release 109.20190905 was used. Raw count data were normalized using Transcripts Per Kilobase Million (TPM), which adjusts for gene length and sequencing depth [15]. Following this, DESeq2 was applied to the normalized count table to identify DEGs, presented as gene names with logFC values for interpretability and downstream analysis [16]. Additionally, the results can be viewed as profile graphs. For this study, DEGs and ncRNAs were screened using threshold values of *p-value* less than 0.05 and logFC values greater than 1 and less than −1. After identifying DEGs, volcano plots were generated for each tissue sample, with the “Enhanced Volcano” package in R.

### 2.3 Screening of non-coding RNA from the datasets

Non-coding RNAs constitute a substantial portion of the human genome. Among them, microRNAs (miRNAs) and long intergenic non-coding RNAs (lincRNAs) are the most common and are prevalently studied under different pathological conditions. Moreover, these proteins are increasingly recognized as critical therapeutic targets and key players in mechanisms underlying both drug sensitivity and resistance [17]. Following the differential expression analysis, miRNAs and lincRNAs were manually selected from the DEG list for further investigation. Similar to the thresholds applied to DEGs, miRNAs, and lincRNAs with a *p-value* less than 0.05 and logFC greater than 1 and less than −1 were prioritized. Additionally, Venn diagrams for both DEGs and ncRNAs across all four cell types were generated using the "VennDiagram" package in R.

### 2.4 Functional enrichment analysis

For the data analysis and functional annotation of DEGs, the significantly differentially expressed genes were submitted to the DAVID database (Database for Annotation, Visualization and Integrated Discovery). DAVID is a very popular and widely used bioinformatics web server tool for analyzing and interpreting gene lists. It offers a rich collection of extensive knowledge base and a range of functional analysis tools to better understand the roles and functions of genes [18].

Furthermore, the results obtained from DAVID were subsequently assessed based on the KEGG pathways and gene ontology (GO) specifically for the biological process aspect. From the list of biological process GO annotations, functional enrichment specific to SLE pathways and immune responses were highlighted using the master list **(**Supplementary File 1**),** and the resulting genes were used for the derivation of the PPI network and topological analysis.

### 2.5 Network analysis

Protein interaction networks can provide valuable insights into the functional and biological characteristics of cellular systems. Hence, the curated genes obtained from functional annotations were analyzed in the Cytoscape software via the StringApp, which is a Cytoscape plugin that retrieves molecular network information from the STRING and STITCH databases. It also facilitates the identification of functional associations and physical interactions while also supporting enrichment analysis for gene groups [19]. Additionally, Cytoscape is an open-source software tool that enables users to visualize different kinds of networks with various features for importing, filtering, clustering, searching, navigating, and exporting networks [20]. To determine interactions with host proteins the network was further expanded by the “Expand network” option in Cytoscape.

To pinpoint key regulatory components within the network, we employed the CytoHubba plugin in Cytoscape. This approach facilitated the identification of "hub genes", distinguished by their extensive interactions with other genes, indicating their potential significance in driving essential biological processes. CytoHubba offers multiple scoring methods for centrality analysis, including degree, closeness, and betweenness centrality. After evaluating these, we selected maximal clique centrality (MCC) as it is known to robustly capture essential nodes within both high- and low-degree regions of the network, reflecting the importance of both densely and sparsely connected proteins in biological systems. The top 10 genes ranked by MCC were identified as hub genes, indicating their potential importance in network stability and function.

The MCC value for a given node *v*, is calculated as:

MCC(*v*) = ∑C ∈ S(*v*) (|C|−1)!

where S(*v*) represents the collection of maximal cliques containing *v*, and (|C|-1)! is the product of all positive integers less than |C|. Additionally, MCC(*v*) is simply equal to its degree, when there is no edge connecting the neighbors of node *v* [21].

We constructed separate networks for both upregulated and downregulated genes and derived their hub genes. However, for further analysis, we focused solely on hub genes obtained from the upregulated DEGs. This focus prioritizes genes likely driving biological changes in SLE, which aligns better with our research on understanding and potentially targeting the complex molecular mechanisms underlying the disease.

### 2.6 Retrieval of Transcription factors and non-coding RNAs associated with hub genes

Transcriptional regulation, a fundamental process for cellular development, responses to genetics and stimuli, and environmental changes, is predominantly controlled by transcription factors (TFs). These proteins interact with specific DNA sequences, referred to as cis-regulatory elements, to activate the transcription of target genes. Alterations in particular TFs, can lead to aberrant expression of the target genes and their downstream pathways, frequently contributing to the development of various diseases. Consequently, understanding the TF-target interaction is essential for comprehending the regulatory networks that drive complex traits and diseases. For this purpose, the TRRUST database, (Transcriptional Regulatory Relationships Unraveled by Sentence Based Text Mining), was utilized. It is a comprehensive, literature-curated, and highly accurate database to identify the different TF-target regulatory interactions of reference genes [22]. The TFs regulating the previously identified hub genes across different cell types were systematically identified from the database.

Additionally, a transcription factor–miRNA coregulatory network was created based on the identified hub genes via the Network Analyst database for the different cell types. The database can be used to analyze gene-expression data, and visualize it, and also contains integrative approaches for PPI network analysis. Furthermore, gene regulatory networks such as TF-miRNAs are crucial for inferring casual relationships in molecular interactions from various knowledgebases [23].

### 2.7 Correlation analysis

For the correlation analysis, a subset that included all the obtained hub genes, transcription factors, and ncRNAs was created for each specific cell type. This subset was subsequently used to derive shared elements from a large gene-expression PBMC dataset (GSE164457 [24]) that comprises 480 samples. The count data from the supplementary file of this dataset were used for the analysis. For the construction of gene regulatory networks and to assess the correlation between genes, the Spearman correlation coefficient was calculated using the “corrplot” package with R software, and a threshold of above 0.5 was set. Different correlation plots were generated for neutrophils, dendritic cells, T cells, and B cells.

### 2.8 Multiple regression analysis

The construction of an MVR model was carried out via a combinatorial approach to create multigene panels consisting of hub genes, TFs, and ncRNAs, that can predict the likelihood of high and low responses to different hub genes. For the analysis, a subset of the abovementioned genes was generated and subsequently used to derive the shared elements from a large gene-expression PBMC dataset (GSE164457) that included 480 samples. The coefficient of determination R^2^ values were also noted for all the hub genes. The primary objective of multivariate regression (MVR) is to establish a connection or correlation between independent and dependent variables. The typical structure of the MVR model can be expressed as follows:

Y = β_0_ + β_1_x_i_ + ϵ_i_

where Y is the dependent variable; β_0_ and β _1_ are the intercept and regression parameters of the model respectively, x is the independent variable, i represents the number of independent variables and ϵ is the standard error. The analysis was performed with the “lm” function in R software [25]. Additionally, added-variable plots (AV plots) were generated for all the cell-type-specific samples with the “avPlots” function in R software. AV plots are scatterplots that reveal the partial correlation between an independent variable and the dependent variable. In the model, the regulators were treated as the dependent variables, while the independent variables included other genes derived from the hub genes and ncRNAs. This approach was useful because there were multiple independent variables in the model and we wanted to understand the specific effect of each one individually.

### 2.9 Construction of gene regulatory networks

A gene regulatory network elucidates the interactions and regulatory relationships among genes [26]. This integrated approach, combining GRN construction, pathway analysis, cis-regulatory element identification, and PPI exploration, enables the iterative refinement of gene networks and uncovers regulatory mechanisms, further enhancing our understanding of the molecular interactions driving cellular responses in different cell types [27]. This approach provides valuable insight into the molecular mechanisms governing biological processes, uncovering how genes interact and organize into functional modules to drive cellular activities [28]. Using the results obtained from correlation plots and regression models, different gene regulatory modules were created for all 4 different cell/tissue samples, i.e., neutrophils, dendritic cells, T cells, and B cells. Additionally, key regulators were identified within these networks, highlighting their critical roles in shaping the cellular responses and functions of each cell type.

### 2.10 Identification of key regulators and ncRNAs

The gene names were individually assigned to the Network Analyst database to identify the non-coding RNAs associated with the regulators identified from the GRNs (IRF3, IRF7, STAT1, MYB, and FOXO1). Moreover, a heatmap was generated with the existing ncRNAs obtained from network analysis and the ncRNAs obtained from DEGs.

### 2.11 Disease–gene network

To conduct a more in-depth assessment of the genes IRF3, IRF7, STAT1, MYB, and FOXO1 and their potential connections to other diseases, a disease-gene network was constructed using DisGeNET and visualized by Cytoscape. The DisGeNET is a platform that comprehensively incorporates vast information from different sources about disease-associated genes and variants [29].

### 2.12 GWAS data analysis

The integration of transcriptomic data analysis with genome-wide association studies (GWAS) helps us to link genetic variation to molecular mechanisms involved in gene activity, providing a deeper understanding of disease mechanisms [30]. To combine gene expression transcriptomics data with GWAS data; significant SNPs were identified from the GWAS Central database. It is a publicly available database with comprehensive resources for the discovery and comparison of phenotype and genotype data from GWAS [31]. In the database “Systemic Lupus Erythematosus” was used as the keyword for deriving the data. To screen the data, abstracts and detailed lists of the studies were downloaded, and the titles and abstracts were read. Studies specific to lupus and lupus-related diseases were taken for further analysis. Using the GWAS tool CATLOUGE, a list of SNPs and other information were collected. A total of 1062 genes were obtained along with their respective variants. From these, 20 genes that were reported for more than 25% of the SNPs were selected for pathway and network analysis. The Pathway analysis was performed with ClueGO, a Cytoscape plugin that is used to perform functional enrichment analysis of genes to determine their potential functions.

### 2.13 Single-cell RNA-seq validation

Peripheral blood mononuclear cell (PBMC) and skin, dermal, and epidermal samples from patients with SLE and controls were retrieved from the Gene Expression Omnibus (GEO) database (Table 5). The obtained samples were analyzed by using the Seurat (v5.0.2) package in R. Primarily, the cells were filtered by using a few quality control metrics such as filtering low-quality cells and empty droplets that contained very few genes, and cell doublets that could yield relatively high gene count. Dying cells or low-quality cells often exhibit extensive amounts of mitochondrial genes. These genes were calculated using the PercentageFeatureSet(1) function and the cells that had fewer than 5% of mitochondrial counts were filtered along with the cells that had genes fewer than 200 and more than 2500 genes to exclude empty droplets and doublets respectively. Next, the data were normalized using the NormalizeData(1) function, and highly variable features were identified using the FindVariableFeatures(1) function. Furthermore, the data were scaled using the ScaleData(1) function, followed by principal component analysis (PCA) to explore patterns within the scaled data. The data were subsequently visualized in various forms such as heatmaps, principal component analysis (PCA), and elbow plots. Furthermore, the control and SLE samples were integrated, and the batch correction was performed using Harmony (v1.2.0). UMAP dimensionality reduction was performed using the first 30 principal components. Later, the nearest neighbors were calculated using FindNeighbors(1) followed by clustering using the FindClusters(1) function [32]. The cell markers that defined clusters were found by differential expression using the FindAllMarkers(1) function. It identifies the markers of a single cluster in comparison to all other cells. Moreover, cells were automatically classified with the obtained markers using SingleR, and cell annotation was performed with the Monaco Immune Database [33]. To analyze the expression levels of different genes, functions such as FeaturePlots(1) and VlnPlots(1) were utilized. These generated feature plots and violin plots are graphical representations of the gene expression data.

## 3 Results

### 3.1 Retrieved differentially expressed genes and ncRNAs

**Fig. 2:**
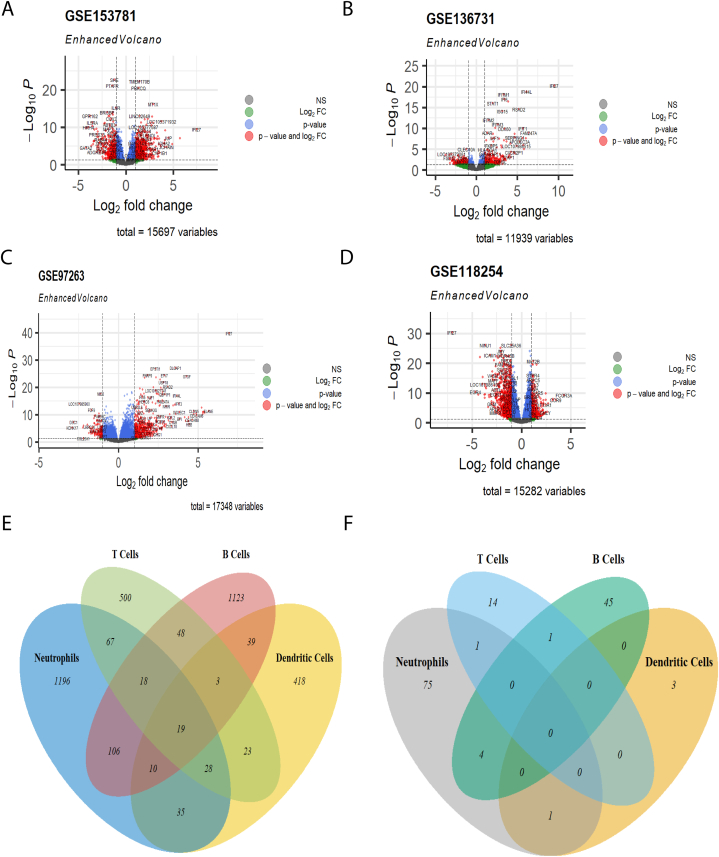
**Differentially expressed genes and non-coding RNAs in different immune cells.** Volcano plots of upregulated and downregulated genes in (A) neutrophils; (B) dendritic cells; (C) T cells; and (D) B cells; Green points depict thedifferentially expressed genes with log2FC value greater than 1 and less than -1, red points depict the significantly differentially expressed genes with log2FC value greater than 1 and less than -1 and *p* values less than 0.05 and the blue points depict the genes that are not significantly differentially expressed. Venn diagrams of (E) differentially expressed genes from the four tissues and, (F) differentially expressed non-coding RNA from the four cell/tissue.

Datasets of SLE for each of the different tissue/cell samples were used for the retrieval of differentially expressed genes and screening of non-coding RNA. The differential expression analysis conducted using GEO2R identified a total of 15,697 genes for neutrophils, 11,939 genes for dendritic cells, 17,348 genes for T cells, and 15,282 genes for B cells. The threshold values used for analysis were a *p-value* less than 0.05 and a logFC value greater than 1 and less than -1 **(**Supplementary File 2**)**. A total of 901 upregulated and 578 downregulated genes were found in neutrophils, while 361 upregulated and 214 downregulated genes were found in dendritic cells. For the T-cells dataset, there were 574 upregulated and 133 downregulated genes. Finally, for B cells, there were 425 upregulated and 941 downregulated genes (Table 2). Volcano plots were generated for the DEGs in the 4 cell types (Fig. 2A-D). A Venn diagram was created for the obtained DEGs, in which 19 genes were common to all 4 cell-type-specific samples namely, IFI27, FAM247A, USP18, FAM247D, LGALS3BP, ISG15, IFI44, RSAD2, IFI44L, HERC5, CMPK2, LOC102723458, EPSTI1, IFI6, OASL, XAF1, IFIT3, ACSL1, and CCL4 (Fig. 2E). Most of these genes were interferon-induced genes (IFI27, ISG15, IFI44, RSAD2, IFI44L, HERC5, IFI6, OASL, XAF1, and IFIT3), genes that regulate the immune system (FAM247A, USP18, FAM247D, LGALS3BP, CMPK2, and ESOTI1), and gene with inflammatory function (CCL4) and lipid metabolism (ACSL1). A unique trend was observed, in which all 18 genes except CCL4 were upregulated in neutrophils, dendritic cells, and T cells, whereas in B cells they were downregulated. Taken together, these findings show that interferons play crucial roles in cells of different cell types.

**Table 2:** Screening of differentially expressed genes and non-coding RNA from tissue-specific datasets.

Dataset	Cells/Tissue	DEGs		DEGs ncRNAs	
		**Up**	**Down**	**Up**	**Down**
GSE153781	Neutrophils	901	578	62	19
GSE136731	cDC2 dendritic cells	361	214	2	1
GSE97263	T cells	573	133	16	0
GSE118254	B cells	425	941	11	39

From the list of DEGs, miRNAs, and lincRNAs were screened manually **(**Supplementary File 3**)**. For neutrophils, we identified 62 upregulated and 19 downregulated ncRNAs while for dendritic cells only 2 upregulated and 1 downregulated ncRNA were found. T cells had 17 upregulated ncRNAs, while B cells had 11 upregulated and 39 downregulated ncRNAs (Table 2). A Venn diagram was also created for the ncRNAs (Fig. 2F), where LINC00294 was common in neutrophils and dendritic cells, LINC00487 was common between neutrophils and T cells and MIR223 was common in T and B cells. Additionally, MIR6821, MIR6891, MIR9-1HG, and LINC01963 were common to neutrophils and B cells.

### 3.2 Functional enrichment analysis and module screening

The functional enrichment analysis performed by DAVID provided functional annotation of the GO biological processes of the genes (Supplementary File 4**)** which were curated based on the master list and immune system responses. The top curated enriched GO terms for neutrophils were “JAK-STAT cascade”, “type I interferon signaling pathway”, “positive regulation of type I interferon production”, “positive regulation of interleukin-17 production”, “positive regulation of neutrophil chemotaxis”, “cellular response to extracellular stimulus”, “response to virus”, and “innate immune response”. In dendritic cells, the top enriched and curated GO biological processes were “positive regulation of RIG-I signaling pathway,” “ISG15-protein conjugation”, “positive regulation of response to cytokine stimulus”, “type I interferon signaling pathway”, and “antigen processing and presentation of exogenous peptide antigen via MHC class II”. For T-cells after curation, the top enriched biological processes were “chemokine-mediated signaling pathway”, “interleukin-27-mediated signaling pathway”, “positive regulation of interferon-beta production”, “T-cell chemotaxis”, “T-cell costimulation”, “positive regulation of T-cell proliferation”, “cellular response to interferon-gamma”, and “cellular response to interferon-gamma”, “T-cell activation”, “JAK-STAT cascade” and “CD4-positive, alpha-beta T-cell activation”. For B-cells, the top curated GO biological processes were “B-cell proliferation involved in immune response,” “immunoglobulin production,” “inflammatory response”, “immune response”, “positive regulation of NF-kappaB transcription factor activity”, and “cellular defense response”. These GO specific to each cell type emphasize key immune processes including interferon signaling, cytokine regulation, antigen presentation, chemotaxis, cell activation, and responses to extracellular and viral stimuli. The gene ontologies of these biological processes related to SLE were highlighted and the corresponding genes were used for protein-protein interaction (PPI) network construction and analysis.

Additionally, pathway analysis was conducted using the KEGG pathways derived from the functional enrichment analysis of the DAVID output (Supplementary File 5**)**. Some of the important pathways found for neutrophils included “Systemic lupus erythematosus”, “Neutrophil extracellular trap formation”, “RIG-I-like receptor signaling pathway”, and “JAK-STAT signaling pathway”. For dendritic cells, a few of the KEGG pathways related to SLE were associated with the “Cytosolic DNA-sensing pathway”, “Herpes simplex virus 1 infection”, “Epstein-Barr virus infection”, “RIG-I-like receptor signaling pathway”, “NOD-like receptor signaling pathway”, “Toll-like receptor signaling pathway”, “Human papillomavirus infection”, “NF-kappa B signaling pathway”, “Apoptosis”, “Human cytomegalovirus infection”, and “Viral life cycle - HIV-1”. A few of the KEGG pathways associated with the functional enrichment of T-cells upregulated genes included “cytokine-cytokine receptor interaction”, “Viral protein interaction with cytokine and cytokine receptor”, “chemokine signaling pathway”, “systemic lupus erythematosus”, “Epstein-Barr virus infection”, “JAK-STAT signaling pathway”, and “cytosolic DNA-sensing pathway”. KEGG analysis of B-cells, including the full dataset and subsets of activated naïve B cells and antigen-secreting cells, identified several key pathways including “chemokine signaling pathway”, “cytokine-cytokine receptor interaction”, “complement and coagulation cascades”, “systemic lupus erythematosus”, “NF-kappa B signaling pathway”, “TNF signaling pathway”, “JAK-STAT signaling pathway”, and “FoxO signaling pathway”. Furthermore, KEGG pathway analysis highlights critical immune-related pathways, including cytokine signaling, JAK-STAT signaling, chemokine interactions, DNA-sensing pathways, and associations with autoimmune.

### 3.3 PPI and topological network analysis

**Fig. 3:**
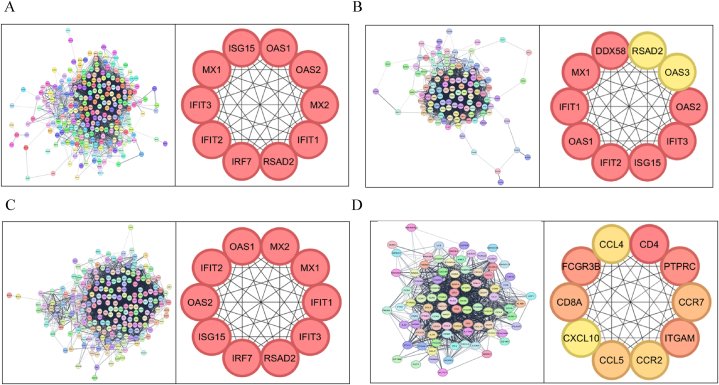
Protein–protein interaction (PPI) Network and Hub Genes. The figure illustrates the PPI networks for immune cell types, highlighting the key hub genes involved in molecular interactions: (A) Neutrophils; (B) Dendritic Cells; (C) T cells; and (D) B cells.

From the curated list of genes, a protein-protein interaction network was created for each cell type, i.e., neutrophils, dendritic cells, T cells, and B cells. For the neutrophil network, there were 3539 edges and 274 nodes; for the dendritic cell network, there were 2135 edges and 127 nodes. The T-cell network had 227 nodes and 3735 edges; and for the B-cell network, there were 1183 edges and 107 nodes. From these topological networks, the hub genes were derived for each tissue sample using the CytoHubba plugin in Cytoscape. Most of the hub genes were interferons (*OAS1, MX1,* and *IFIT3*), interferon-stimulated genes (*ISG15*, and *RSAD2*) and receptor molecules (*TLR4, CD86,* and *PTPRC*). In neutrophils, the hub genes retrieved from the network analysis by the MCC algorithm were *OAS1/2, MX1/2, IFIT1/2/3, RSAD2, IRF7,* and *ISG15* (Fig. 3A). Most of these genes are activated by type I interferons (IFN-α and *IFN-β*) and contribute to disease flares. For dendritic cells, *RSAD2, OAS1/2/3, IFIT1/2/3, ISG15, MX1,* and *DDX58* were the derived hub genes (Fig. 3B). These genes are mainly stimulated by IFN and ISGs, and also the dysregulation of *DDX58* is known to contribute to the overproduction of these interferons. Moreover, the hub genes of T-cells include *OAS1/2, MX1/2, IFIT1/2/3, RSAD2, IRF7,* and *ISG15* these genes are also interferon-stimulated genes that contribute to inflammation and tissue damage (Fig. 3C). In B-cells, the hub genes obtained are *CCL4, CD4, PTPRC, CCR7, ITGAM, CCR2/5, CXCL10, CD8A,* and *FCGR3B* (Fig. 3D). These genes play major roles in B-cell activation, adhesion, and antibody production. Given the observed divergence in DEGs within the B-cell dataset compared to the literature, we opted for a more granular analysis. We subsequently stratified the B-cell samples into activated naïve B cells and antigen-secreting cells (ASCs) for independent gene expression profiling. The hub genes of activated naïve B cells included *STAT3, IL1A, IL1B, CD4, IL6, IL10, IFNG, TNF, STAT1,* and *NFKB1* (Supplementary Figure 1A), and ASCs hub genes included, *IL6, IL10, CD4, IL1B, CD86, CD40, IFNG, TNF, CD8A,* and *CD80* **(Supplementary** Figure 1B**)**. The hub genes of naïve B cells emphasize cytokine signaling and immune activation while antigen-secreting cells’ hub genes highlight immune regulation and antigen presentation.

**Fig. 4:**
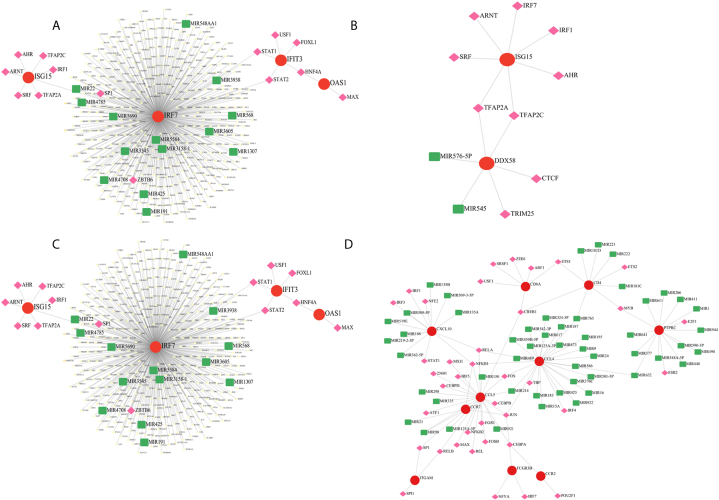
Transcription Factor (TF)-miRNA Coregulatory Network The figure displays the TF-miRNA coregulatory networks for different immune cell types: (A) Neutrophils; (B) Dendritic cells; (C) T cells; and (D) B cells. In each network, red circles denote the different hub genes of the cells, pink diamonds denote the transcription factors and the green squares denote the ncRNAs.

The key regulators of these genes were retrieved from the TRRUST database **(Supplementary** File 6**)**. Also, a TF-miRNA coregulatory network was created using the network analyst database with the hub genes obtained for each cell type, and they were visualized using Cytoscape (Fig. 4 A-D). These networks reveal the key interactions between the hub genes and the different ncRNAs and regulators.

### 3.4 Correlation analysis

**Fig. 5:**
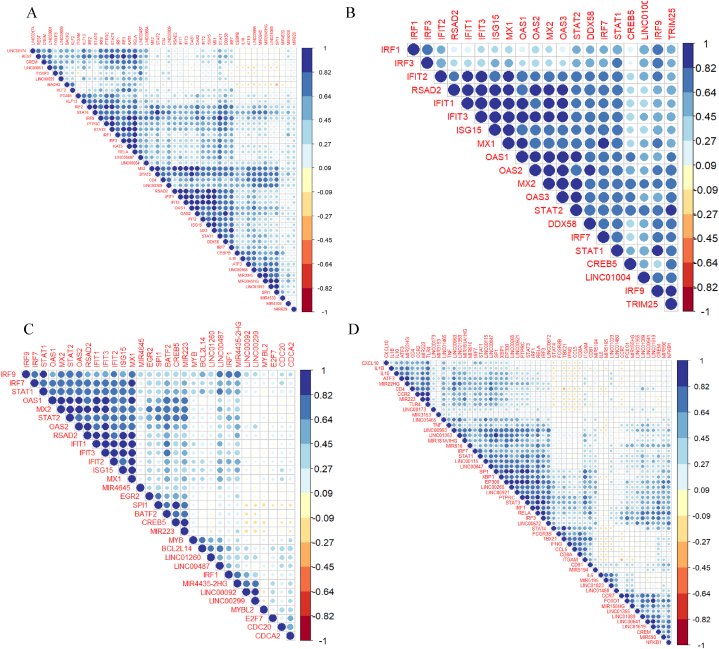
Heatmap depicting correlation between hub genes and regulators The figure shows correlation plots for hub genes, key transcription factors, and non-coding RNAs (ncRNAs) in immune cells: (A) Neutrophils; (B) Dendritic cells; (C) T cells; and (D) B cells.

To deduce the relationships between the obtained hub genes from section 3.3, the regulators from the TRRUST database, and the screened ncRNAs, correlation analysis was conducted and the results are shown in heatmaps (Fig. 5). In neutrophils, *DDX58*/RIG-I and the hub gene *IFIT3* highly correlate with IFN-stimulating transcription factor *IRF1/2*/and the hub gene *IRF7* along with the regulators *STAT1* and 2, and IFNs such as *MX1/2, OAS1/2*, and *RSAD2*. Furthermore, *IRF3*, and *ISG15* which are hub genes, and other IFN hub genes were correlated with *STAT1*. The lncRNA LINC00968 was correlated with IFN-related hub genes such as *OAS1, MX2*, and regulators like *IRF7*, a hub gene, and *STAT1/2/6,* while LINC00487 was correlated with *IRF3, STAT1, IRF7/9,* and IFN-stimulated hub genes – *IFIT1/2* (Fig. 5A). In dendritic cells, the expression of the hub gene *DDX58*/RIG-I strongly correlated with the ubiquitin ligase enzyme TRIM25 which induces RIG-I-mediated IFN – beta production [34], the transcription factor *IRF9*, the IFN stimulated hub genes *MX1/2, OAS1/2/3,* and *RSAD2* and with *STAT1/2*. Moreover, the ncRNA LINC1004 was strongly correlated with the *STAT2*, *MX2, IRF3/9, TRIM25*, and IFN-stimulating genes (Fig. 5B). In T cells, we found that all the interferons and ISGs were strongly correlated with each other and with regulators such as the hub genes *IRF7, IRF1*, and *STAT1/2*. The *BATF2* transcription factor correlated with MIR223, and this miRNA correlated with most of the interferons as well. Furthermore, *MYB* was correlated with BCL2L14, LINC01260, and LINC00487 and was slightly correlated with a few ISGs such as the hub genes *ISG15, MX1*, and *IFIT3*. LINC00487 shows a high correlation with STAT1 (Fig. 5C). Finally, the correlation plot for B cells showed that *FOXO1* was strongly correlated with hub gene CCR7, a chemokine receptor molecule, the ncRNAs MIR590, and LINC01619. Additionally, *XBP1* correlated with *STAT3*, *PTPRC* and *EP300* (Fig. 5D). Moreover, a correlation plot was generated to determine the relationships between interferons (IFNs), different regulators, ncRNAs, and transcription factors (Supplementary Fig. 2). FOXO1 was positively correlated with LINC00641 and LINC01619 with a correlation coefficient of 0.5. Additionally, *EBF1* and *PAX5* exhibited a high correlation. The correlation analysis highlights the central role of IFN-related genes across all cell types, emphasizing their association with SLE.

### 3.5 Regression analysis

Multivariant regression analysis (MVR) revealed associations among hub genes from section 3.3, their regulators, and non-coding RNAs across different immune cell types (Table 3). For neutrophils, *IRF7* exhibited high-scoring MVR models for the ncRNAs LINC00487, LINC00861, and LINC01004, and IFN-stimulated genes, like *MX1, RSAD2*, and *ISG15*, with other regulators such as STAT1/2. Furthermore, LINC00968 is highly associated with many interferon-stimulated hub genes such as IFIT1/3, MX1/2, OAS1/2, and RSAD2 with R^2^ values greater than 0.95. In dendritic cells, the hub gene DDX58/RIG-I showed the highest-scoring MVR model for OAS2/3, IFIT1, and STAT2 with a score of 0.8618. TRIM25 also yielded high R^2^ values of 0.9686 and 0.9411 for *OAS2* and *OAS1*, respectively. *STAT1/2* exhibited high-scoring MVR models for many of the hub genes, namely, *DDX58, OAS1/2, IFIT1/3*, and *ISG15*. The majority of T-cell hub genes achieved the highest-scoring MVR model, with an R^2^ value exceeding 0.9. IRF7 had high scores for *STAT2, SPI1, RSAD2, MX1, ISG15, IFIT1,* and *CREB5* with a high R^2^ value of 0.8855. STAT regulators were found to have a high score in genes such as *MX2, IFIT3, RSAD2, IRF7, ISG15,* and *OAS2*. The *MYB* transcription factor also significantly affected *BCL2L14, IRF1*, LINC00487, LINC01260, and MIR223 with an R^2^ value of 0.5297. Finally, the MVR model for B cells showed a high score for TBX21, as did hub genes such as *CD4, CCR2, CCR7,* and *CCL5*, and LINC00847 which yielded a high R^2^ value of 0.8512 for *CD8A*. *CD4* also had a high scoring value for LINC00963.

**Table 3:** Multivariate regression model for associated hub genes.

HUB GENES	R^2^ VALUE	HIGHLY SIGNIFICANT GENES	P- VALUE
**NEUTROPHIL**			
**IFIT3**	0.9833	ATF3, IFIT1, IFIT2, LINC00487, LINC00968, MIR4530, MX2, OAS2, RSAD2, STAT2, STAT3	<2.2e-16
**IRF7**	0.9393	ATF3, CEBPB, IFIT1, IL10, ITF1, IRF1, KLF2, LINC00174, LINC00487, LINC00861, LINC00937, MIR17HG, MIR548Q, MIR550A1, MX1, NFKBIA, PTPRC, RELA, RSAD2, STAT1, STAT2	<2.2e-16
**RSAD2**	0.9715	DDX58, IFIT1, IFIT2, IFIT3, IRF7, ISG15, LINC00582, LINC00937, LINC00968, MIR550A1, OAS1, OAS2, STAT2	<2.2e-16
**IFIT1**	0.9664	CD4, IFIT1, IRF2, LINC00428, LINC00664, LINC00487, LINC00968, MIR3945HG, MIR4530, MX1, OAS2, RSAD2, STAT1, STAT6	<2.2e-16
**MX1**	0.9635	DDX58, IFIT1, IL10, IRF2, IRF7, IRF9, ISG15, KLF13, LINC00487, LINC00861, LINC00968, LINC00937, MIR4308, MIR4440, MX2, OAS1, STAT6, VDR	<2.2e-16
**MX2**	0.9772	ATF3, IFIT3, IFIT2, IRF2, ISG15, KAT5, LINC00402, LINC00487, LINC00582, LINC00664, LINC00937, LINC00968, LINC01366, MIR4530, MX1, OAS2, STAT1, STAT2	<2.2e-16
**ISG15**	0.9483	IRF9, LINC00487, LINC00659, LINC00664, MIR4441, MIR550A1, MIR624, MX1, MX2, RSAD2, STAT2	<2.2e-16
**OAS1**	0.9632	CD4, IFIT1, IRF2, LINC00428, LINC00664, LINC00487, LINC00968, MIR3945HG, MIR4530, MX1, OAS2, RSAD2, STAT1, STAT6	<2.2e-16
**IFIT2**	0.9381	ATF3, IFIT3, KLF13, LINC00174, LINC00309, LINC00582, LINC00659, MIR4530, MIR624, MX2, OAS2, RSAD2, STAT3	<2.2e-16
**OAS2**	0.9788	IFIT1, IFIT2, IFIT3, IRF2, ITGAM, KLF2, LINC00487, LINC00968, LINC00664, LINC00937, MIR550A1, MX2, OAS1, RSAD2, STAT1	<2.2e-16
**DENDRITIC CELLS**			
**DDX58**	0.8618	CREB5, IFIT1, OAS2, OAS3, STAT2	<2.2e-16
**RSAD2**	0.9529	IFIT1, IFIT2, IFIT3, ISG15, OAS1, OAS2	<2.2e-16
**OAS3**	0.9494	DDX58, IRF9, LINC01004, MX2, OAS2	<2.2e-16
**OAS2**	0.9686	CREB5, DDX58, IFIT1, IFIT2, ISG15, LINC01004, OAS1, OAS3, RSAD2, STAT1, TRIM25	<2.2e-16
**IFIT3**	0.9732	CREB5, IFIT1, IFIT2, OAS1, RSAD2, STAT2	<2.2e-16
**ISG15**	0.9126	MX1, OAS2, RSAD2, STAT1, STAT2	<2.2e-16
**IFIT2**	0.8967	IFIT3, IRF1, OAS2, RSAD2	<2.2e-16
**OAS1**	0.9411	CREB5, IFIT3, MX1, OAS2, RSAD2, STAT1, STAT2, TRIIM25	<2.2e-16
**IFIT1**	0.9431	CREB5, DDX58, IFIT3, OAS2, RSAD2, STAT2	<2.2e-16
**MX1**	0.9298	IRF1, IRF9, ISG15, MX2, OAS1	<2.2e-16
**T CELLS**			
**OAS1**	0.9506	IFIT3, MIR223, MX1, OAS2, RSAD2, SPI1	<2.2e-16
**MX2**	0.9671	BCL2L14, CDC20, EGR2, IRF9, ISG15, LINC00487, MX1, OAS2, STAT1, STAT2	<2.2e-16
**MX1**	0.9514	BATF2, CREB5, IFIT1, IFIT3, IRF7, IRF9, ISG15, LINC00092, MX2, OAS1, OAS2, SPI1	<2.2e-16
**IFIT1**	0.9589	BCL2L14, IFIT3, IRF7, ISG15, MX1, RSAD2	<2.2e-16
**IFIT3**	0.9777	IFIT1, IFIT2 IRF1, LINC00092, LINC00487, MX1, OAS1, OAS2, RSAD2, STAT2	<2.2e-16
**RSAD2**	0.9586	IFIT1, IFIT2, IFIT3, IRF7, LINC00092, OAS1, OAS2, SPI1, STAT2	<2.2e-16
**IRF7**	0.8844	STAT2, SPI1, RSAD2, MX1, ISG15, IFIT1, CREB5	<2.2e-16
**ISG15**	0.9329	CDC20, CDCA2, IFIT1, IRF7, MX1, MX2, STAT1, STAT2	<2.2e-16
**OAS2**	0.967	IFIT2, IFIT3, IRF1, LINC00092, LINC00299, LINC00487, LINC01260, MX1, MX2, MYBL2, OAS1, RSAD2, SPI1, STAT1, STAT2	<2.2e-16
**IFIT2**	0.9134	IFIT3, IRF1, LINC00092, MIR4435.2HG, OAS2, RSAD2, SPI1	<2.2e-16
**B CELL**			
**CD4**	0.928	EP300, ITGAM, LINC00963, LINC01465, MIR155HG, MIR22HG, MIR5195, PTPRC, SP1, TBX21	<2.2e-16
**CD8A**	0.7949	IFNG, ITGAM, LINC00847, MIR155HG, PTPRC, STAT4, TLR4	<2.2e-16
**PTPRC**	0.9669	CCL5, CD4, CREM, EP300, IRF3, IRF7, LINC00672, STAT1, STAT3, STAT4, TBX21, TLR4	<2.2e-16
**CCR7**	0.9085	CD8A, CREM, ITGAM, STAT3, STAT4, TBX21	<2.2e-16
**FCGR3B**	0.7556	ATF3, CCL5, CD4, CD81, FCGR3B, FOXO1, IL1BIRF3, MIR155HG, MIR22HG, MIR590, SP1, STAT1, STAT3, STAT4, TLR4	<2.2e-16
**CCL5**	0.8511	FCGR3B, IFNG, ITGAM, LINC01353	<2.2e-16
**CXCL10**	0.6808	IL1B, IRF1, STAT1	<2.2e-16
**ITGAM**	0.9488	IL1B, MIR223, MIR22HG, TLR4, XBP1	<2.2e-16
**CCR2**	0.9379	FOXO1, IRF3, LINC01089, STAT4, TBX21	<2.2e-16
**CCL4**	N/A	N/A	N/A

**Fig. 6:**
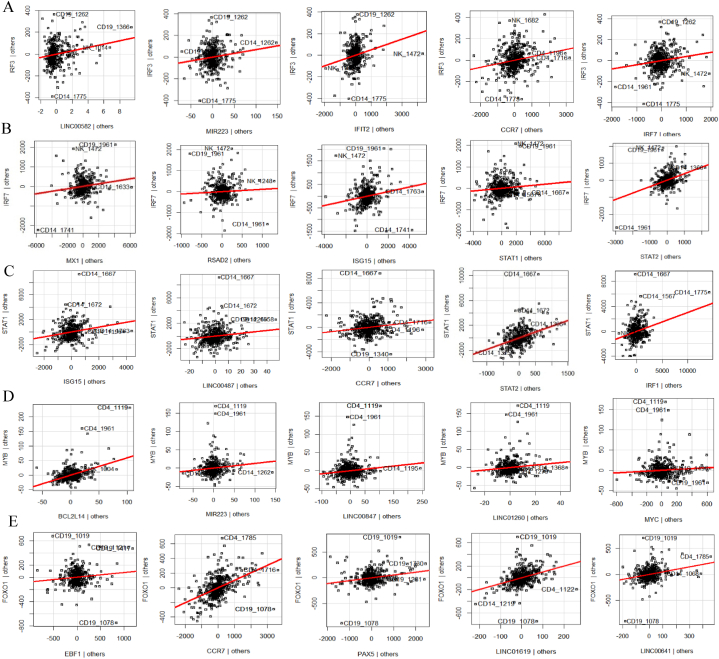
Multivariable Regression Analysis of Key Regulators The figure presents AV scatter plots that demonstrate the performance of multivariable regression models for critical regulatory genes. The plots illustrate: (A) IRF3 (R^2^ = 0.9250461); (B) IRF7 (R^2^ = 0.8532618); (C) STAT1 (R^2^ = 0.9041221); (D) MYB (R^2^ = 0.5947396); (E) FOXO1 (R^2^ = 0.9179705).

MVR models are visualized through AV plots. AV plots for *IRF3* showed a positive correlation with genes such as LINC00582, MIR223, *IFIT2, ISG15,* and *IRF7* with an R^2^ value of 0.9250461 (Fig. 6A). *IRF7* AV plots revealed positive association for *MX1, RSAD2, ISG15, STAT1,* and *STAT2* with an R^2^ value of 0.8532618 (Fig. 6B). STAT1 was positively related to *ISG15*, LINC00487, *OAS2, STAT2,* and *IRF1* with an R^2^ value of 0.9041221 (Fig. 6C). *MYB* was positively related to *BCL2L14, MYC*, MIR223, LINC00847, and LINC01260 with an R^2^ value of 0.5947396 (Fig. 6D). Finally, the B-cell hub genes *CCR2* and *FCGR3B* had one of the highest scoring MVRs for the regulator *FOXO1* with R^2^ values of 0.9086 and 0.9488, respectively. Furthermore, the *FOXO1* AV plots showed positive associations with *CCR7, EBF1, PAX5,* LINC00641, and LINC01619 with an R^2^ value of 0.9179705 (Fig. 6E). Moreover, *EBF1* was positively correlated with PAX5 with an R^2^ value of 0.9150934, while *NFATC1* was positively correlated with *JUNB, CCR7*, and LINC00641 with an R^2^ value of 0.8953587 (Supplementary Figure 3). The MVR models show strong positive correlations between transcription factors and target genes, with high R^2^ values, indicating robust relationships. Key factors like *IRF3, IRF7, STAT1,* and *MYB* are strongly linked to immune-related genes, while *FOXO1* is closely associated with B-cell hub genes and other regulatory factors like *EBF1* and *PAX5*, highlighting their central role in immune regulation.

### 3.6 ncRNAs associated with selected regulators

A heatmap was generated to evaluate the relationships between ncRNAs from the DEG list and the network analyst database from section 2.10, along with regulators identified from the correlation plot and MVR models, namely, *IRF3, IRF7, STAT1, MYB,* and *FOXO1*. **(**Supplementary File 7**)**. The threshold for correlation values in the heatmap was set to ≥0.5 to highlight significant associations. *IRF3* was correlated with LINC001619, LINC01089, LINC00487, LINC1074, and LINC00861, while *IRF7* was strongly correlated with LINC00487 and LINC00847. *STAT1* was correlated with LINC00963 and *FOXO1* was correlated with LINC01089, LINC01619, LINC00847, MIR590, and LINC0174 (Supplementary Figure 4). Several ncRNAs, including LINC00487, LINC00847, LINC01089, and LINC01619, are shared between key regulators like *IRF3, IRF7,* and *FOXO1*, indicating their central role in modulating immune-related transcription factors.

### 3.7 Gene regulatory networks for neutrophils, dendritic cells, T cells, and B cells

**Fig. 7:**
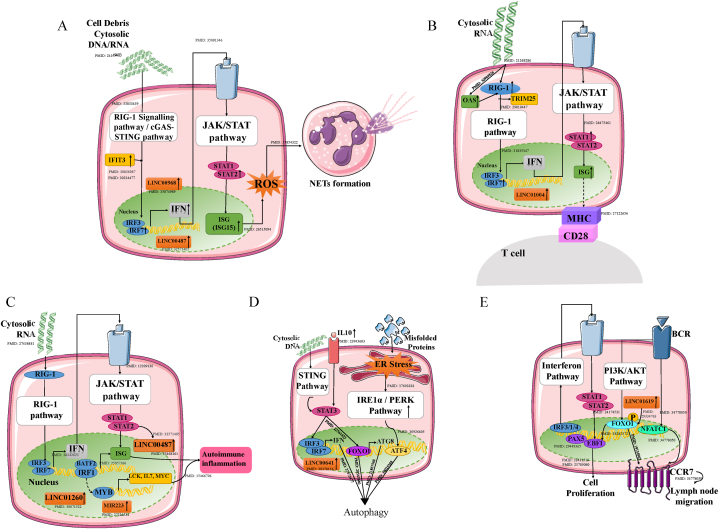
Proposed Gene Regulatory Networks Across Distinct Immune Cell Types The figure illustrates unique gene regulatory networks, constructed based on hub genes, key transcription factors, and ncRNAs for (A) Neutrophils; (B) Dendritic cells; (C) T cells; and (D) Activated naïve B cells (E) Antigen-secreting cells. The upward arrow (↑) indicates that the respective genes or ncRNAs are upregulated. PubMed identifiers (PMIDs) corresponding to the references used are provided. Detailed summary is mentioned in the Supplementary Document 1.

We used insights from sections 3.4, 3.5, 3.6 and a comprehensive literature review to construct cell type-specific gene regulatory networks, involving key transcription factors and their target genes (Fig. 7). Furthermore, cell-specific inductive pathways were traced based on the set endpoints **(**Supplementary Document 1**)**.

For the neutrophil gene network, NETosis is considered the endpoint because it is one of the defense mechanisms used by neutrophils under stress conditions. Moreover, NETs contain antigens that can stimulate autoantibody production [35]. By constructing a gene regulatory network, we also found that IFNs are activated by cytosolic DNA/RNA via the cGAS-STING and RIG-1 pathways and by the regulators IRF3 and IRF7. The interferons further activate interferon-stimulated genes via the JAK/STAT pathway with regulators STAT1/2. While the activation of IFNs and ISGs is well-established, our findings highlight the crucial role of LINC00968 and LINC00487 in mediating this pathway. Additionally, ISG activation leads to ROS production, which in turn promotes NET formation (Fig. 7A). This study not only validates the known connections between IFNs and NETosis but also provides new insights into the pivotal roles of *IRF3, IRF7,* and *STAT1* in this pathway.

The hub genes for dendritic cells were mostly IFNs and ISGs that are produced by the RIG-I and JAK/STAT pathways, respectively, which are mediated by transcription factors such as IRF3/7 and STAT1. Type I interferons are considered effector molecules because they are known to promote the activation and maturation of dendritic cells via autocrine signaling. The effects include increased production of IFNs and upregulation of molecules such as CD80, CD86, and MHC class II on the DC surface, thereby, activating T cells. While the role of IFNs in DC activation and antigen presentation is well-established, our findings highlight the critical involvement of *IRF3, IRF7, STAT1*, and LINC01004 in mediating these processes. Moreover, type I interferons are recognized for their role in directly enhancing B-cell activation, stimulating antibody production, and supporting T-cell survival [36] (Fig. 7B).

Interferon-stimulated genes (ISGs) and MYBs are considered effector molecules of the T-cell gene regulatory network. ISGs are known to drive immune infiltration and contribute to organ damage, as observed in conditions like lupus nephritis [37]. In contrast, MYBs play a central role in the development and maturation of T cells [38] and are also involved in the differentiation of high-affinity B cells and the regulation of immunological memory [39], further influencing adaptive immune responses. MYB regulates the activation of several key genes, including *BCL2L14, LCK, MYC,* and *IL7*, which are associated with immune dysregulation. This dysregulation can result in the generation of autoreactive and proinflammatory cells, ultimately driving B-cell activation and contributing to inflammatory and autoimmune responses. The gene network in accordance with existing literature revealed that interferons are secreted via the RIG-I and JAK/STAT pathways, mediated by key regulators such as *IRF3, IRF7,* and *STAT1*. Additionally, transcription factors like *BATF2* and *IRF1* were identified as critical players, driving immune infiltration and contributing to organ damage. Our study further reveals that transcriptional regulators such as *IRF3, IRF7, STAT1,* and *MYB* are critically involved in T cell-mediated autoimmunity, along with ncRNAs LINC00487, LINC01260, and MIR223 with their expression patterns linked to immune dysregulation. (Fig. 7C).

In activated-naïve B cells, autophagy is considered the endpoint in the gene regulatory network (GRN). Previous research has shown that autophagy is not only a consequence of B cell activation but also a critical mechanism utilized by naïve B cells in systemic lupus erythematosus (SLE). Autophagy is specifically activated in SLE naïve B cells, potentially serving as a survival strategy for autoreactive cells [40]. Additionally, autophagy has been demonstrated to play an essential role in plasmablast differentiation and is activated by cellular stress, such as unfolded proteins in the endoplasmic reticulum (ER), UV radiation, or cytokines like interleukin-10, through various signaling pathways. Our study validates these findings and further highlights the strong correlation between LINC00641, an ncRNA upregulated in SLE, and *FOXO1*, a key regulator of autophagy. Notably, our results emphasize the critical role of *FOXO1* in autophagy regulation within activated-naïve B cells, providing new insights into its importance in the context of SLE (Fig. 7D).

In ASCs GRN lymph node migration and cell proliferation were considered as the endpoints. Previous research has shown that ASCs from SLE patients exhibit increased expression of genes associated with survival, proliferation, and antibody production, including cytokines such as IL-6 and IL-10, adhesion molecules, and components of the B-cell receptor (BCR) signaling pathway. Additionally, ASCs upregulate *CCR7*, a receptor that may play a pivotal role in guiding these cells back to lymph nodes. This migration facilitates further antigen encounters, enhances antibody production, and potentially provides critical survival signals [41]. Moreover, factors such as FOXO1, EBF1, with ncRNA LINC01619 seem to play key roles in regulating these survival, proliferation, and migration pathways in ASCs from SLE patients [42] Our study validates these findings and further reveals the critical involvement of transcription factors such as *FOXO1, EBF1*, and interferons in regulating these pathways (Fig. 7E).

### 3.8 Disease – gene network

**Fig. 8:**
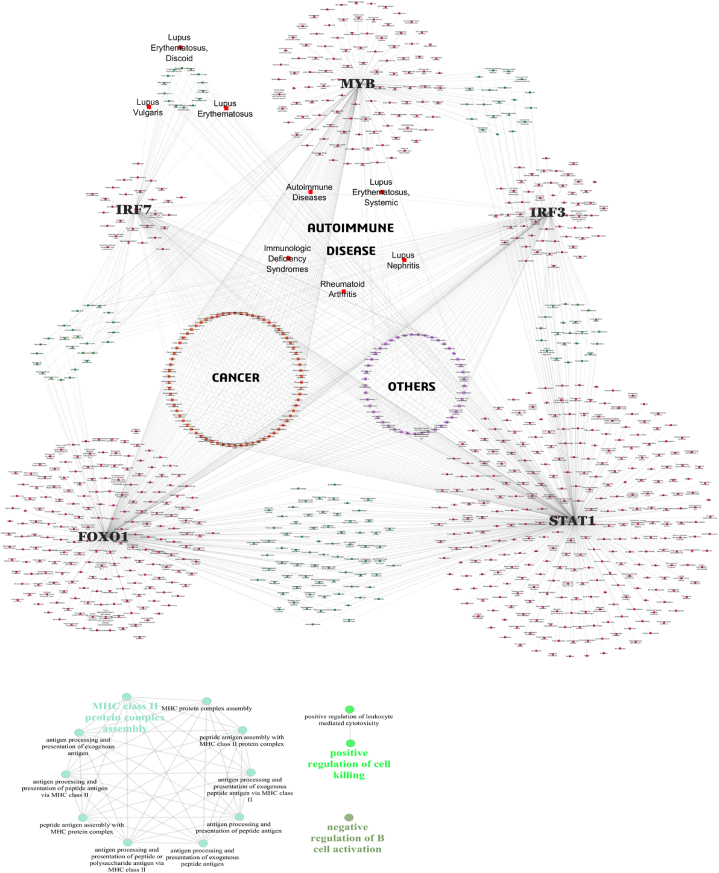
**A) Disease-Gene Network Representation.** The figure illustrates the disease-gene network constructed to identify associations between genetic variants and disease pathways. Nodes represent genes or diseases, with edges signifying known or predicted interactions. **B) GWAS Gene Pathways Identified Using ClueGO in Cytoscape.** The figure depicts enriched biological pathways derived from GWAS-identified genes, analyzed using the ClueGO plugin in Cytoscape.

To explore the broader disease associations of *IRF3, IRF7, STAT1, MYB,* and *FOXO1*, we utilized the DisGeNET database to retrieve the relevant data **(**Supplementary File 8), to construct a comprehensive disease-gene network which was visualized with Cytoscape software (Fig. 8A).

The network revealed shared associations for diseases namely, “Immunologic Deficiency Syndromes” and “Autoimmune Diseases” with the regulators *IRF3, IRF7, STAT1*, and *FOXO1*, moreover, diseases such as “lupus erythematosus, systemic” “lupus nephritis”, and “rheumatoid arthritis” showed a linked to the regulators *IRF3, FOXO1,* and *STAT1*. Diseases like “lupus erythematosus, discoid”, “lupus valgaris”, and “lupus erythematosus” showed a relation to the regulators *STAT1* and *IRF3*. This network revealed not only the shared association with autoimmune diseases but also connections to various other diseases encompassing cancers, and other diseases namely, infectious diseases, diabetes mellitus, asthma, atherosclerosis, Alzheimer's disease, and coronary artery diseases, highlighting the crucial roles of these genes. This network not only validates the established roles of these regulators in autoimmune diseases but also highlights their potential broader involvement in a wide range of pathological conditions.

### 3.9 GWAS exploration

According to the SNP–gene data retrieved from the GWAS Central, 20 genes had at least 25 % of the SNPs. These genes include *UBE2L3*, *TPI1P2*, *LOC407835*, *STAT4*, *ITGAM*, *HLA-DRB1*, *HLA-DQA1*, *HLA-DRA*, *HLA-DRB5*, *HLA-DQB1*, *HLA-DQA2*, *FAM167A*, *BLK*, *BTNL2*, *BANK1*, *TNPO3*, *IRF5*, *LOC100506023*, *OLIGIG3*, and *TNFAIP3* (Table 4) An analysis of these top 20 genes with most SNPs via the ClueGO plugin in Cytoscape revealed three main functional categories “MHC class II protein complex assembly” which accounts for most of the genes (76.92%), suggesting a strong focus on this process. MHC class II molecules play a vital role in presenting antigens to T cells, facilitating immune recognition and response. The next is the “positive regulation of cell killing” which accounts for 15.38% of the total population, suggesting that accelerated cell death and defective clearance can lead to the accumulation of autoantigens and the production of autoantibodies at a later stage of the disease [43]. Finally, the “negative regulation of B-cell activation” which accounts for 7.69% of genes, can lead to the aberrant activation of B cells, contributing to the pathogenesis of the disease [44] (Fig. 8B). Furthermore, no significant genetic variants were found to be associated with the *IRF3, STAT1, MYB,* or *FOXO1* genes except for the *IRF7* gene, which had two SNPs, rs1131665 and rs1061502.Table 5

**Table 4:** Top 20 genes with the maximum number of SNPs.

GENES	No. of SNPs
HLA-DQB1	140
HLA-DRA	115
HLA-DQA2	115
HLA-DRB5	109
HLA-DRB1	105
HLA-DQA1	90
ITGAM	74
UBE2L3	69
STAT4	49
TPI1P2	45
LOC407835	43
BTNL2	37
IRF5	33
TNPO3	30
TNFAIP3	29
BANK1	24
LOC100506023	22
BLK	20
FAM167A	17
OLIG3	15

**Table 5:** Single-cell RNA-Seq data with the accession number for each sample.

GeEO Accession ID	Cells/Tissue	No. Of Samples Selected	Location	Ref. Publ
*GSE162577*	PBMC	1 SLE	China	75
		1 Control		
*GSE179633*	Skin biopsies - Dermal	7 SLE	China	76
		2 Control		
	Skin biopsies - Epidermal	2 SLE		
		2 Control		

### 3.10 Single-cell analysis

**Fig. 9:**
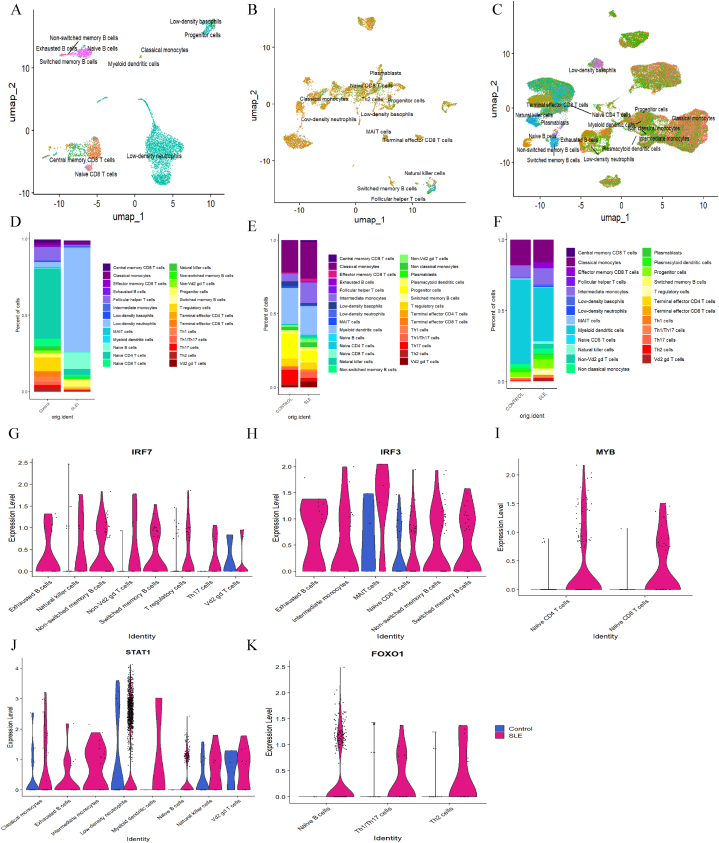
Identification of the regulators in different samples UMAP representation of sc-RNASeq data of different samples, colour coded by clusters (A) PBMC samples, (B) Dermal samples, and (C) Epidermal samples. Cell proposition plot for (D) PBMC samples, (E) Dermal samples, and (F) Epidermal samples. Volcano Plots depicting sc-RNASeq analysis of controls and SLE samples for different regulators in PBMC dataset (G) IRF7 (H) IRF3 (I) MYB (J) STAT1 and (K) FOXO1.

Single-cell RNA-Seq analysis was conducted using the Seurat package in R to validate the expression of key regulators (*IRF3, IRF7, STAT1, MYB,* and *FOXO1*) identified from the gene regulatory networks, specifically to determine their expression across different cell types. The clusters formed for the three different sources of samples, PBMCs, dermal samples, and epidermal samples are given in Fig. 9 A-C.

Moreover, the propositions of the different samples with the different clusters were visualized. Among the PBMCs, low-density neutrophils were more common in the SLE samples. For B cells, switched memory B cells and naïve B cells had more cell propositions (Fig. 9D). In dermal samples, myeloid dendritic cells, classical monocytes, progenitor cells, and follicular helper T cells accounted for a greater percentage of the cells (Fig. 9E). For epidermal samples, classical monocytes, intermediate monocytes, and myeloid dendritic cells had a greater number of cells (Fig. 9F).

Crucial transcription factors and regulators, namely, *IRF3, IRF7, STAT1, MYB,* and *FOXO1*, were analyzed in different samples and different cell clusters. Violin plots illustrating the expression levels of selected targets across all the cell clusters in different samples are given in Supplementary Figs. 5, 6, and 7. Additionally, feature plots depicting the expression levels of regulators *IRF3, IRF7, STAT1, MYB,* and *FOXO1* in different clusters of different samples, namely, PBMCs, and dermal, and epidermal samples are shown in Supplementary Figs. 8, 9, and 10 respectively.

In PBMC samples from individuals with SLE, elevated levels of *IRF7* were detected in various cell types, including exhausted B cells, non-switched memory B cells, natural killer cells, switched memory B cells, non-Vd2 gd T cells, T regulatory cells, Th17 cells, and Vd2 gd T cells (Fig. 9G). Similarly, SLE patient samples showed increased levels of *IRF3* in exhausted B cells, intermediate monocytes, MAIT cells, naïve CD8^+^ T cells, non-switched memory B cells, and switched memory B cells (Fig. 9H). The *MYB* regulator was high in naïve CD4^+^ T cells and naïve CD8^+^ T cells (Fig. 9I). *STAT1* levels were notably high in classical monocytes, exhausted B cells, intermediate monocytes, low-density neutrophils, myeloid dendritic cells, naïve B cells, natural killer cells, and Vd2 gd T cells among the SLE samples (Fig. 9J). The *FOXO1* regulator exhibited relatively greater expression in SLE naïve B cells, Th1/Th17 cells, and Th2 cells (Fig. 9K). *PAX5* was expressed only in naïve B cells, switched memory B cells, non-switched memory B cells, and exhausted B cells. *NFATC1* was expressed in switched memory B cells and a few other cells, whereas, *EBF1*, *CCR7, ATF4,* and *STAT3* were more highly expressed in SLE samples from naïve and switched B cells than in those from other cells (Supplementary Figs. 11 and 12).

**Fig. 10:**
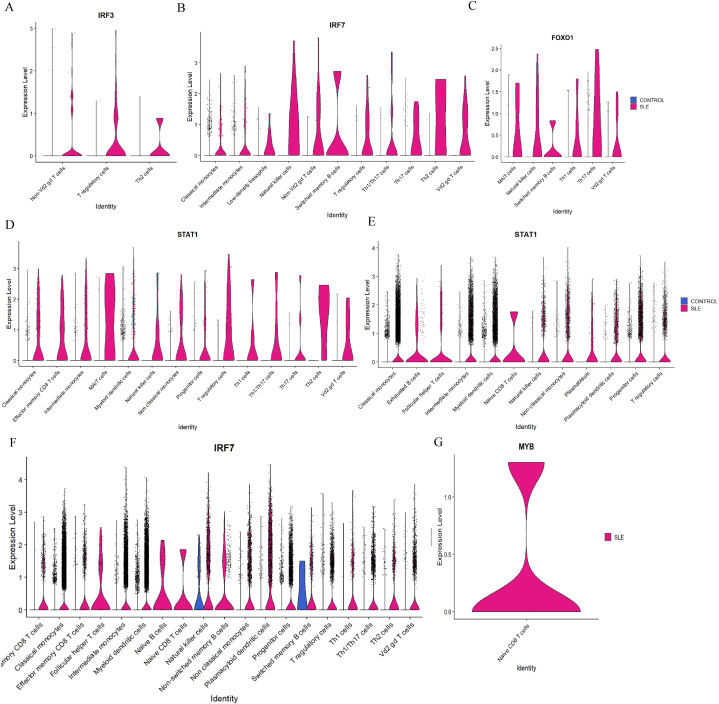
Transcription factors identified from scRNA-seq analysis SLE and control samples across epidermal and dermal immune cell subsets. (A–D) Violin plots representing the expression of IRF3, IRF7, FOXO1, and STAT1 in epidermal immune cell subsets, highlighting significant differential expression between SLE (pink) and control (blue) samples. (E–G) Violin plots illustrating expression patterns of STAT1, IRF7, and MYB in dermal immune cell subsets.

In the study of epidermal samples, high levels of specific transcription factors were primarily found in certain cell clusters from SLE patients, while control samples showed almost no expression of these regulators**.** For *IRF3*, increased expression was observed in non-Vd2 gd T cells, T regulatory cells, and Th2 cells. (Fig. 10A). For *IRF7*, high expression was observed in classical monocytes, intermediate monocytes, low-density basophils, natural killer cells, non-Vd2 gd T cells, switched memory B cells, T regulatory cells, Th1/Th17 cells, Th17 cells, Th2 cells, and in Vd2 gd T cells (Fig. 10B). The *FOXO1* regulator was highly expressed in several cell types, including MAIT cells, natural killer cells, switched memory B cells, Th1 cells, Th17 cells, and Vd2 gd T cells in patients with SLE (Fig. 10C). Similarly, for *STAT1*, effector memory CD8 T cells, intermediate monocytes, classical monocytes, MAIT cells, myeloid dendritic cells, natural killer cells, non-classical monocytes, progenitor cells, T regulatory cells, Th1 cells, Th1/Th17 cells, Th17 cells, Th2 cells, and Vd2 gd T cells exhibited high expression levels for SLE samples (Fig. 10D). In contrast, no *MYB* expression was found in any cell clusters. Regulators like *STAT3, ATF4, IRF4, EBF1, CCR7,* and *EIF2AK3* were seen to have high expression in switched memory B cells and few other clusters (Supplementary Figs. 13 and 14).

In dermal samples, for the *STAT1* gene, most cells were expressed only in SLE samples, for instance, classical monocytes, exhausted B cells, follicular helper T cells, intermediate monocytes, myeloid dendritic cells, naïve CD8 T cells, natural killer cells, non-classical monocytes, plasmablasts, plasmacytoid dendritic cells, progenitor cells, and T regulatory cells were found (Fig. 10E). Additionally, *IRF7* gene expression was detected in clusters of patients with SLE, namely, central memory CD8 T cells, classical monocytes, effector memory CD8 T cells, follicular helper T cells, intermediate monocytes, myeloid dendritic cells, naïve B cells, naïve CD8^+^ T cells, natural killer cells, non-switched memory B cells, non-classical monocytes, plasmacytoid dendritic cells, progenitor cells, switched memory B cells, T regulatory cells, Th1 cells, Th1/Th17 cells, Th2 cells, and Vd2 gd T cells (Fig. 10F). Finally, MYB expression was detected only in naïve CD8^+^ T cells (Fig. 10G). Moreover, the violin plots of regulators namely, *IRF4, IRF1, ATF4, CCR7,* and *STAT2* across different B-cell clusters and other cells are shown in Supplementary Figs. 15 and 16. Overall, the analysis reveals that key transcription factors such as *IRF3, IRF7, STAT1, MYB*, and *FOXO1* are widely expressed across various immune cell types in SLE patients, underscoring their crucial roles in immune dysregulation. These regulators are prominently found in one or more of the cell types, including B cells, T cells, dendritic cells, and neutrophils, highlighting their potential involvement in the pathogenesis of SLE and offering insights for potential therapeutic targeting.

## 4 Discussion

Systemic lupus erythematosus (SLE) is a chronic autoimmune disorder in which interferons play a pivotal role in driving disease progression. Studies have demonstrated that genes of the interferon signature such as *IFIT1, RSAD2, IFI27, ISG15,* and *RNASE2* are related to the severity of the disease [45]. Furthermore, elevated levels of interferons are also observed in various systemic autoimmune rheumatic diseases (SARDs), including systemic sclerosis (SSc), mixed connective tissue disease (MCTD), Sjögren's syndrome, undifferentiated connective tissue disease (UCTD), and idiopathic inflammatory myopathies (IIM) [46]. Despite extensive insights into interferon pathways and immune cell interactions, the precise molecular landscape specific to individual cell types remains elusive, hindering a comprehensive view of its pathogenesis. To address this, our study explores the unique molecular interactions within the distinct immune cell types – neutrophils, dendritic cells, T cells, and B cells – to construct comprehensive gene regulatory networks. Analysis of these GRNs revealed overexpression of interferons and interferon-stimulated genes, indicating the activation of critical transcription factors: interferon-regulatory factors *IRF3* and *IRF7*, along with the signal transducer STAT1. Additionally, T cells showed marked activation of the *MYB* regulator, while in B cells, the transcription factor *FOXO1* was positively associated with mediators of *CCR7* and *EBF1* production, as well as autophagy processes, ultimately facilitating antibody production.

*IRF3* and *IRF7* are implicated in various diseases, including cancer [47], autoimmune disorders [48], inflammatory diseases [49], cardiac hypertrophy [50], psoriasis [51], bacterial infections [52], and atherosclerosis [53]. Moreover, targeting them offers a promising therapeutic approach. For instance, auranofin inhibits *IRF3* [54] while curcumin [55] and scoparone [56] are known to suppress *IRF3*. Moreover, Aryl hydrocarbon receptor-interacting protein (AIP) [57] and *ATF4* [58] are known to suppress viral-induced type I IFN by inhibiting *IRF7* activation. *STAT1* is another critical transcription factor implicated in diseases like recurrent aphthous stomatitis (RAS) [59], inflammatory diseases [60], antitumor therapies [61], and liver fibrosis [62]. Natural compounds like THIF (4′,5,7-trihydroxyisoflavone [63]), and EGCG (epigallocatechin gallate) [64] have been shown to regulate the *STAT1* signaling pathway. Similarly, modulation of *IRF3* and *STAT1* by statins has been linked to reduced IFN and ISG expression [65]. Furthermore, *MYB* is considered a potential target for anticancer therapy [66] for acute myeloid leukemia (AML), T-cell acute leukemia (T-ALL), adenoid cystic carcinoma (ACC), and other types of cancer [67,68], and also for other diseases like inflammatory osteoclast formation and bone resorption [69]. Natural compounds like plumbagin and celastrol also suppress *MYB* target genes [70]. Lastly, *FOXO1* is seen to have potential involvement in diseases like esophageal adenocarcinomas [71], type 2 diabetes mellitus, Parkinson's disease [72], metabolic syndrome [73], cardiovascular diseases, aging, and stem-cell activity [74]. Inhibition of *FOXO1* through drugs like AS1842856 and trifluoperazine has shown promise in reducing blood sugar [75], impairing autophagy in diabetes [76], preventing left ventricular atrophy [77], and overcoming chemoresistance in breast cancer [78].

In SLE, immune cell dysregulation drives disease progression through intricate signaling pathways. Initially, it is known autoantigen exposure activates naïve neutrophils, leading to interferon (IFN) production and the induction of IFN-stimulated genes (ISGs). This IFN-induced environment produces excessive reactive oxygen species (ROS), eventually leading to NETosis. Several studies show that elevated NET formation and reduced NET degradation expose modified autoantigens that stimulate autoantibody production and exacerbate tissue damage leading to SLE [79,80]. This was further proved in our analysis, dictating the formation of NETosis through central transcriptional regulators—*IRF3, IRF7,* and *STAT1*—play key roles in this process. Moreover, ncRNAs such as LINC00968, a key regulator for NF-kappaB signaling pathways [81] and LINC00487 which is strongly induced by IFNα [82] further support the disease progression.

The autoantigens exposed either by neutrophils or by EBV enhance IFN signaling in dendritic cells (DCs), promoting its maturation [36]. The mature DCs subsequently migrate to lymphoid organs, where they prime T cells through IFN and ISG-mediated pathways. Additionally, our study demonstrates that the regulatory genes *IRF3, IRF7,* and *STAT1,* along with the regulatory ncRNA LINC01004, are critical in modulating these inflammatory pathways.

Furthermore, studies say that CD4^+^ T cells are activated through antigen-presenting cells like dendritic cells [83] or via RIG-I pathway signaling [84], which induces IFN production and stimulates ISGs [83]. These pathways are activated by key regulators such as *IRF3, IRF7*, and *STAT1*, which promote immune cell infiltration and contribute to organ damage, as seen in lupus nephritis inflammation [[85], [86], [87]]. Additionally, our study identified MYB, a central transcription factor in T and B cell maturation, that activates inflammatory genes such as *BCL2L14, LCK, MYC,* and *IL7*, as a target for T cells. Additionally, ncRNAs such as MIR223 which promotes type I IFN production [88], LINC00487 that regulates interferon regulation [82], and LINC01260 that is regulated by the NF-κB pathway [89] together enhance T-cell hyperactivation, and lead to autoantibody production and tissue damage.

Research shows that activated naïve B cells in SLE exhibit unique transcriptional responses to endoplasmic reticulum (ER) stress caused by misfolded protein accumulation, which triggers autophagy as a cellular coping mechanism [90,91]. Autophagy not only supports B cell activation but also plays a critical role in Plasmablast differentiation [92]. Through careful analysis and validation, we found that regulators including *IRF3, IRF7, FOXO1*, and the ncRNA LINC00641 underscore the role of autophagy in sustaining B cell function in SLE. Finally, antigen-secreting cells drive lymph node migration and cellular proliferation, potentially leading to a significant increase in antibody production. This process is regulated by key factors including *IRF3, STAT1,* and *FOXO1* with the ncRNA LINC01619 likely modulating these pathways. Collectively, these immune cell interactions and regulatory networks reveal the complex pathogenesis of SLE, underscoring potential therapeutic targets for modulating immune responses in the multifaceted autoimmune disorder (**For a detailed explanation, please refer to Supplementary Document 1).**

This complex network of immune interactions highlights potential therapeutic targets for SLE. Specifically, targeting *FOXO1* and *MYB* in later disease stages could help mitigate organ damage and autoantibody production by modulating T and B cell hyperactivation. In early SLE stages, where lymphocyte activation is minimal, therapeutic strategies focusing on myeloid immune cells, such as dendritic cells and neutrophils, may effectively reduce interferon and ISG levels. This stage-specific approach aligns with SLE's evolving immune landscape, offering a potentially nuanced and precise therapy [93].

Additionally, cell-type-specific drug delivery systems (DDSs) present promising advantages, including minimal immunogenicity, low cytotoxicity, extended circulation, targeted delivery, and high biocompatibility [94]. These systems facilitate targeted drug delivery to diseased cells, potentially improving treatment effectiveness and minimizing side effects compared to conventional methods. They also align with precision medicine principles by customizing treatments based on individual patient profiles [95,96].

Our findings align with existing literature by confirming the critical roles of *IRF3, IRF7*, and *STAT1* in SLE pathogenesis, while providing novel insights into their cell-type-specific activities. Notably, we identified *MYB's* role in T cell maturation and *FOXO1's* involvement in naïve B cell autophagy and ASCs, highlighting previously underexplored regulatory mechanisms. Additionally, we uncovered the contributions of ncRNAs such as LINC00487, LINC00968, LINC01004, LINC01260, MIR223, LINC00641, and LINC01619 in modulating interferon-driven inflammation and disease progression, offering a fresh perspective on non-coding elements in SLE. These cell-specific regulatory networks advance current understanding and identify actionable targets for stage-specific, precision therapies. While this study provides valuable insights into gene regulatory networks, it may not fully capture the complexity of biological processes. Additionally, the potential off-target effects of the identified therapeutic targets were not assessed. Future research can include *in vivo* validation to confirm the therapeutic potential and specificity of these targets.

Despite these limitations, our robust computational pipeline—integrating systems biology approaches—demonstrates the importance of targets identified from gene regulatory networks relevant to SLE pathology across multiple immune cell types, including neutrophils, dendritic cells, T cells, and B cells. This analysis was achieved using in silico approaches such as correlation analysis, multivariate regression, and single-cell RNASeq analysis. These methods reveal the critical role of these targets in disease activation and progression, addressing inherent constraints in studying SLE's complex pathogenesis.

## 5 Conclusion

In conclusion, this study provides a detailed analysis of the gene regulatory networks involved in SLE pathogenesis across immune cell types, including neutrophils, dendritic cells, T cells, and B cells. Key transcription factors such as *IRF3, IRF7, STAT1, MYB*, and *FOXO1*, along with associated non-coding RNAs, play pivotal roles in driving interferon signaling, immune cell activation, and autoantibody production. Targeting these molecular regulators, particularly in a cell-type-specific manner, offers promising therapeutic strategies for modulating immune responses in SLE and potentially other autoimmune diseases. Our systems biology approach provides valuable insights into the complex mechanisms of SLE, opening avenues for precision medicine-based therapies.

## CRediT authorship contribution statement

**Blessy Kiruba:** Writing – original draft, Visualization, Investigation, Formal analysis, Data curation. **Akshayata Naidu:** Writing – original draft, Methodology, Investigation, Formal analysis, Data curation, Conceptualization. **Vino Sundararajan:** Writing – review & editing, Supervision, Project administration. **Sajitha Lulu S:** Writing – review & editing, Supervision, Project administration, Conceptualization, Methodology.

## Lead contact

Further information and requests for resources should be directed to and will be fulfilled by the lead contact, Dr. Sajitha Lulu S (ssajithalulu@vit.ac.in)

## Data availability statement

The data supporting the findings of this study are available within the main article and supplemental information.

## Funding statement

This research did not receive any specific grant from funding agencies in the public, commercial, or not-for-profit sectors.

## Declaration of competing interest

The authors declare that they have no known competing financial interests or personal relationships that could have appeared to influence the work reported in this paper.
