# Supplementary material for: Mapping integral cell-type-specific interferon-induced gene regulatory networks (GRNs) involved in systemic lupus erythematosus using systems and computational analysis
Source: Heliyon. 2024 Dec 18;11(1):e41342. doi: 10.1016/j.heliyon.2024.e41342 (PMC11751531; doi:10.1016/j.heliyon.2024.e41342)
Supplement: Multimedia component 9 [file mmc9.pptx]

## Slide 1
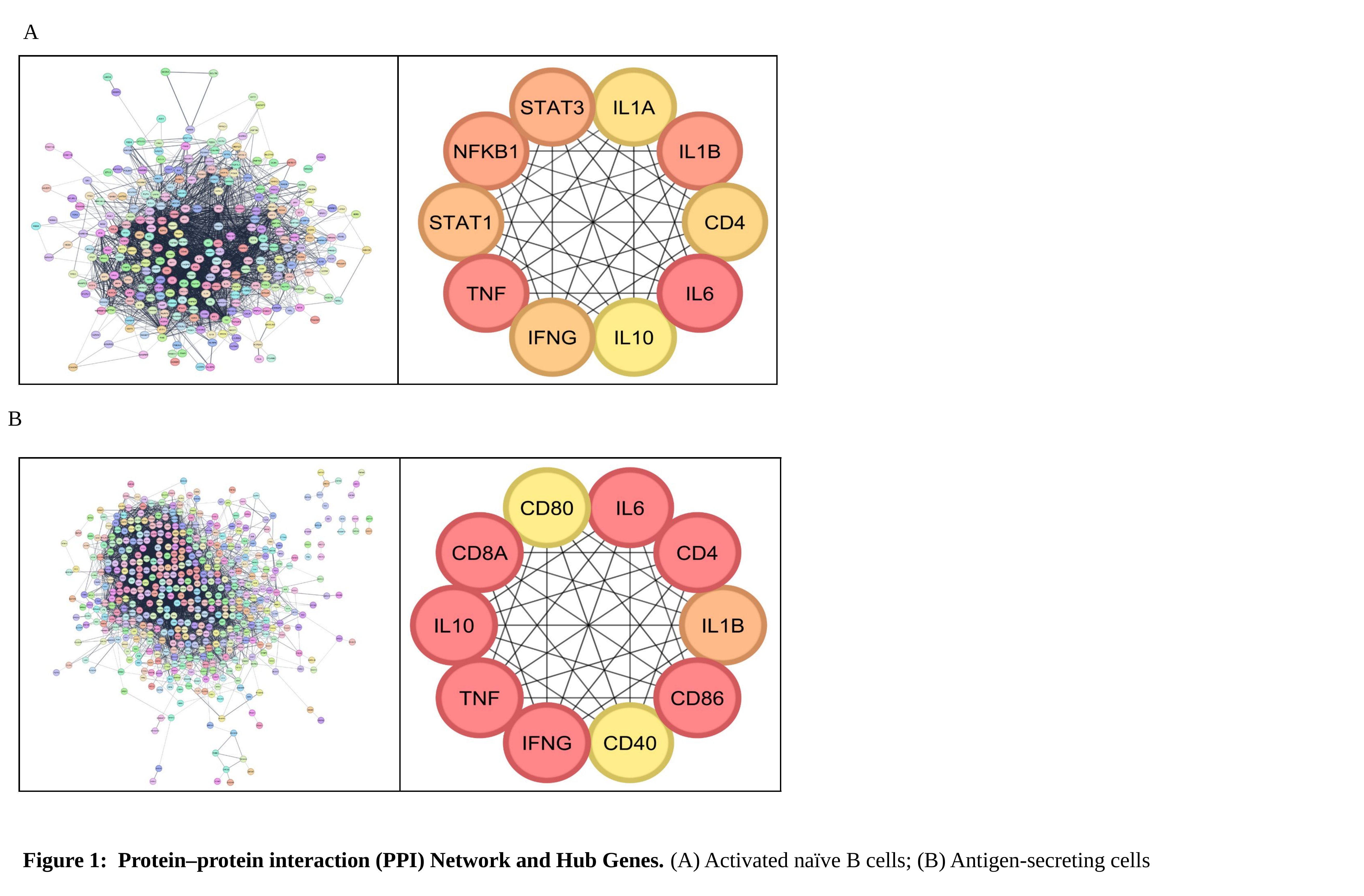

A
| | |
| --- | --- |
B
| | |
| --- | --- |
Figure 1: Protein–protein interaction (PPI) Network and Hub Genes. (A) Activated naïve B cells; (B) Antigen-secreting cells

## Slide 2
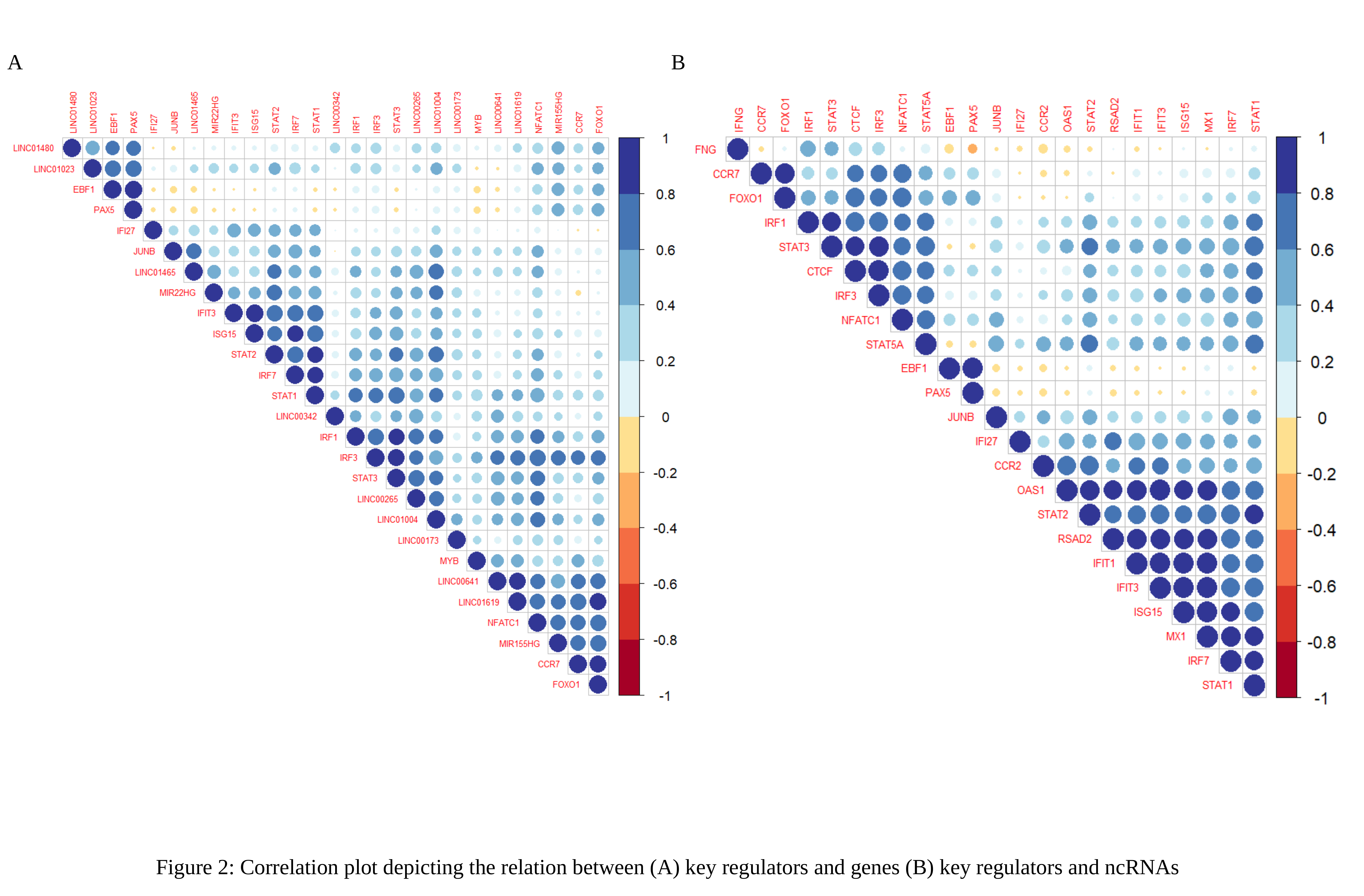

A
B
Figure 2: Correlation plot depicting the relation between (A) key regulators and genes (B) key regulators and ncRNAs

## Slide 3
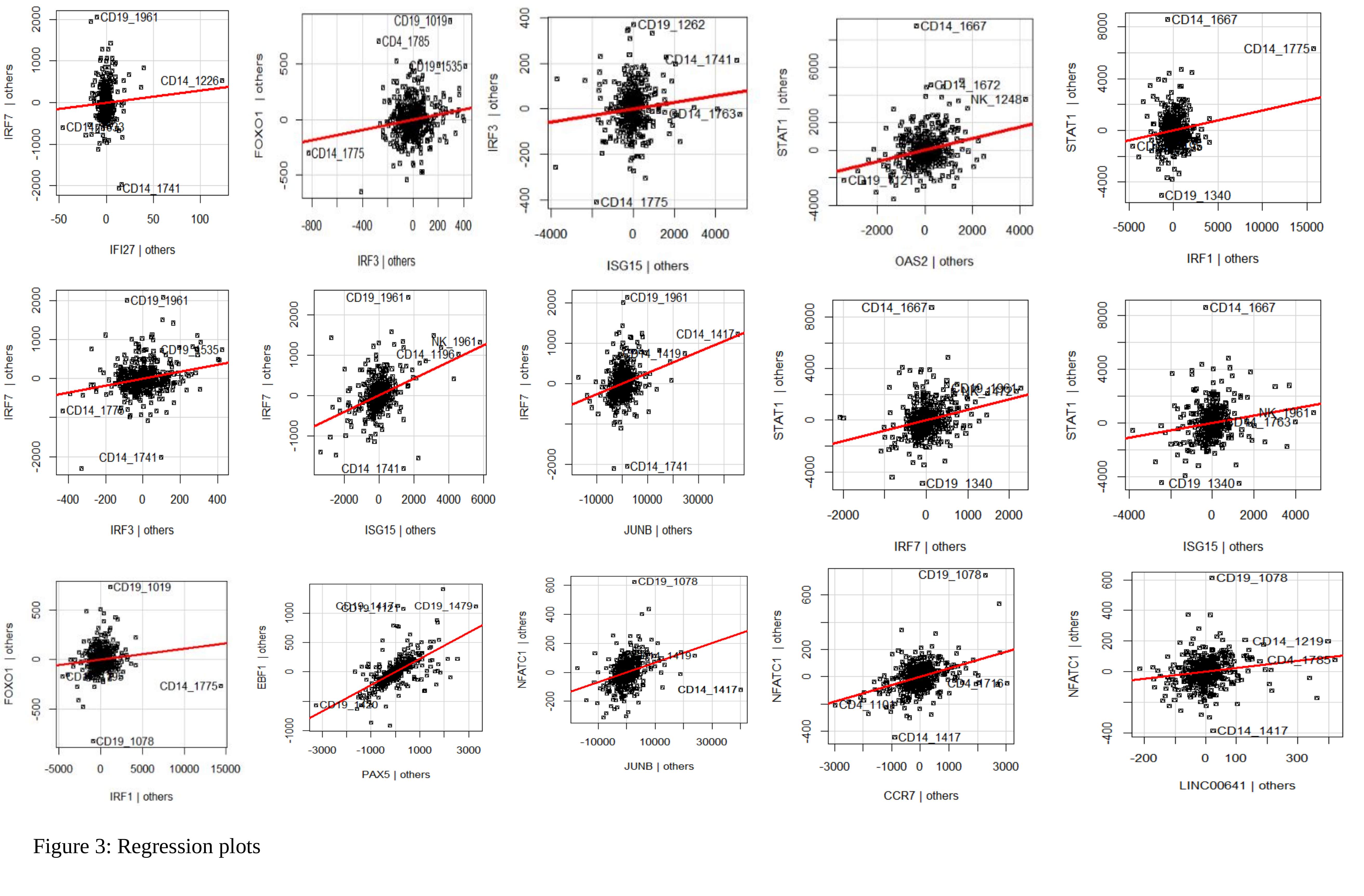

Figure 3: Regression plots

## Slide 4
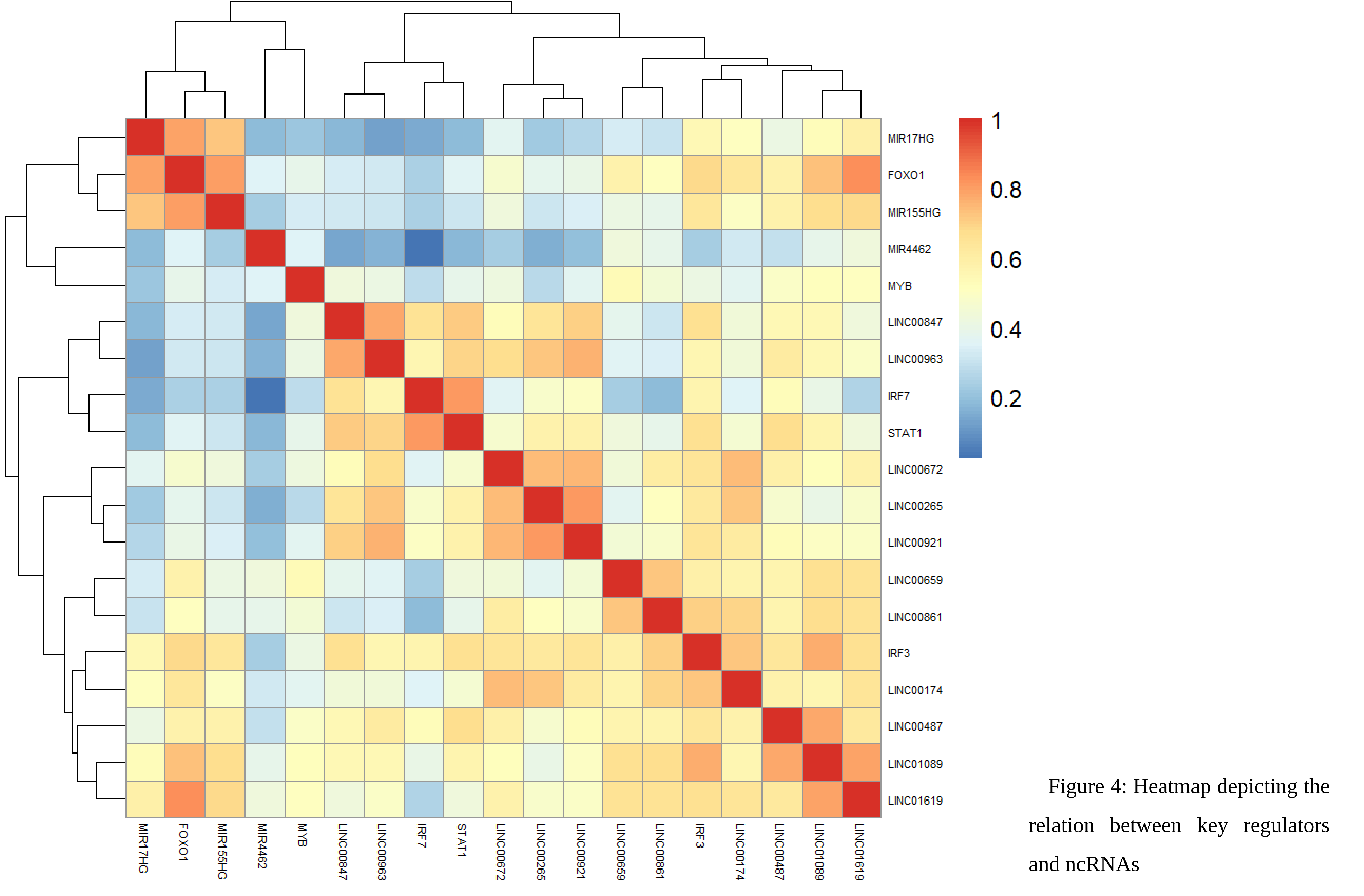

Figure 4: Heatmap depicting the relation between key regulators and ncRNAs

## Slide 5
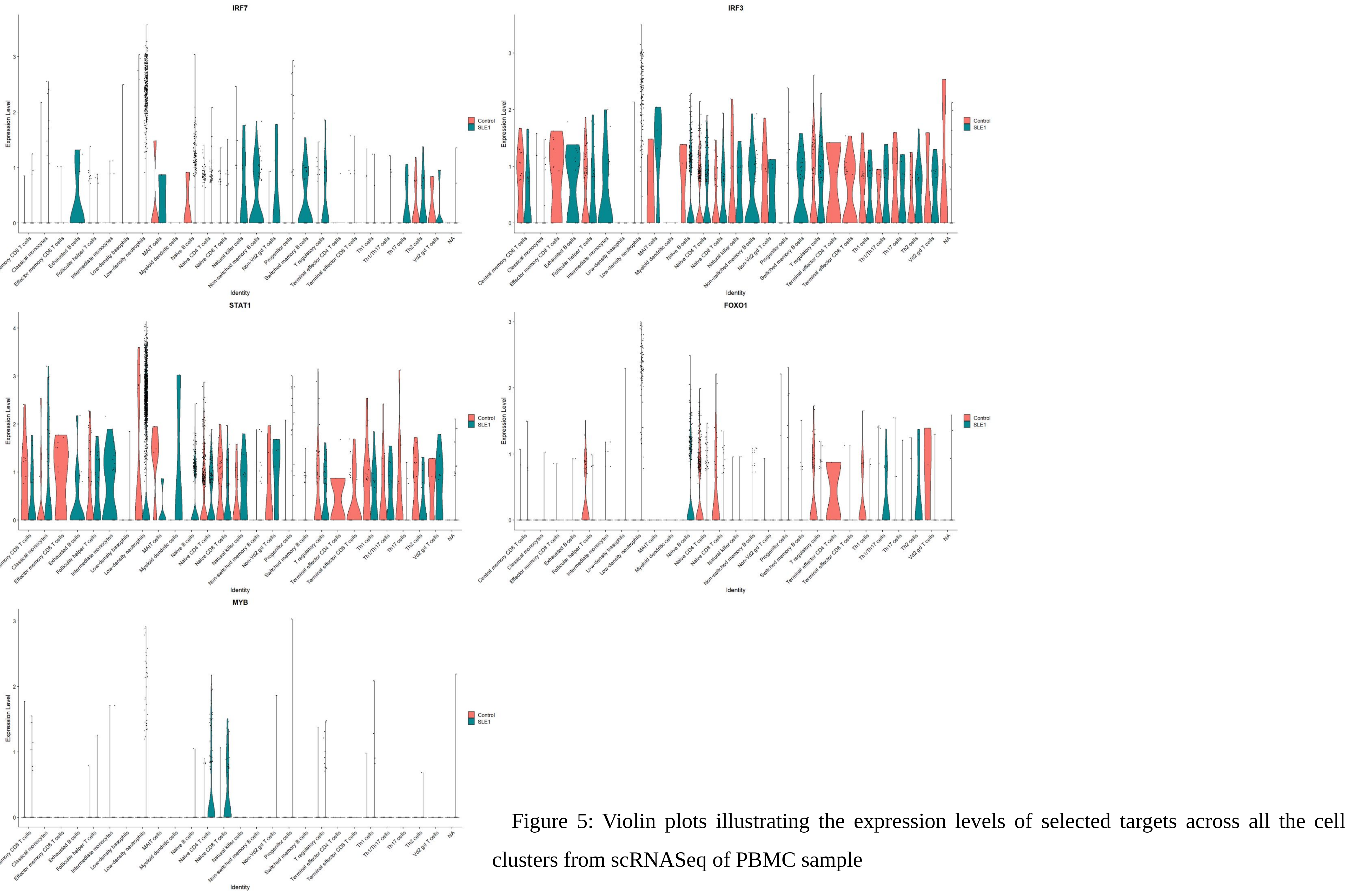

Figure 5: Violin plots illustrating the expression levels of selected targets across all the cell clusters from scRNASeq of PBMC sample

## Slide 6
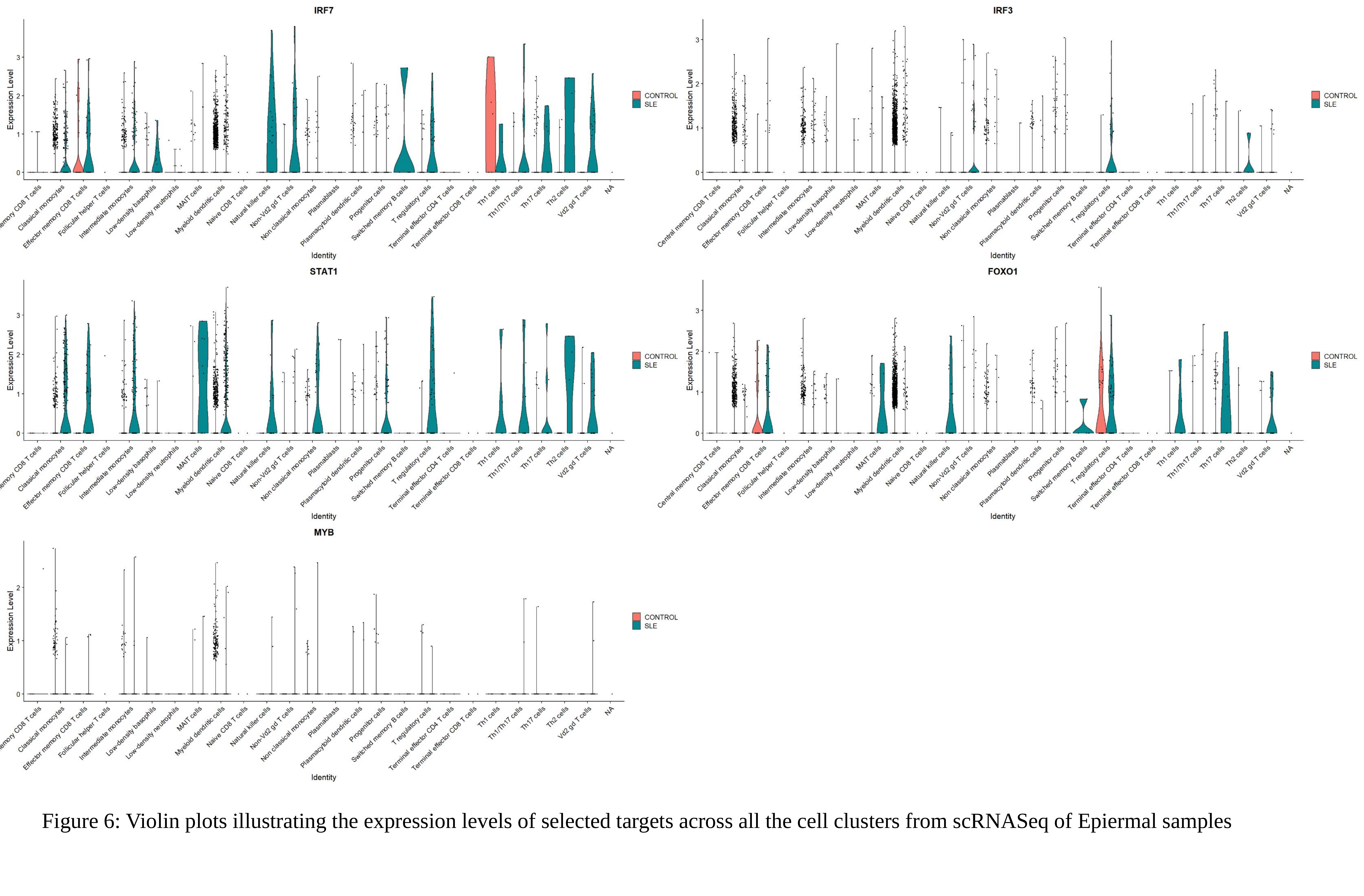

Figure 6: Violin plots illustrating the expression levels of selected targets across all the cell clusters from scRNASeq of Epiermal samples

## Slide 7
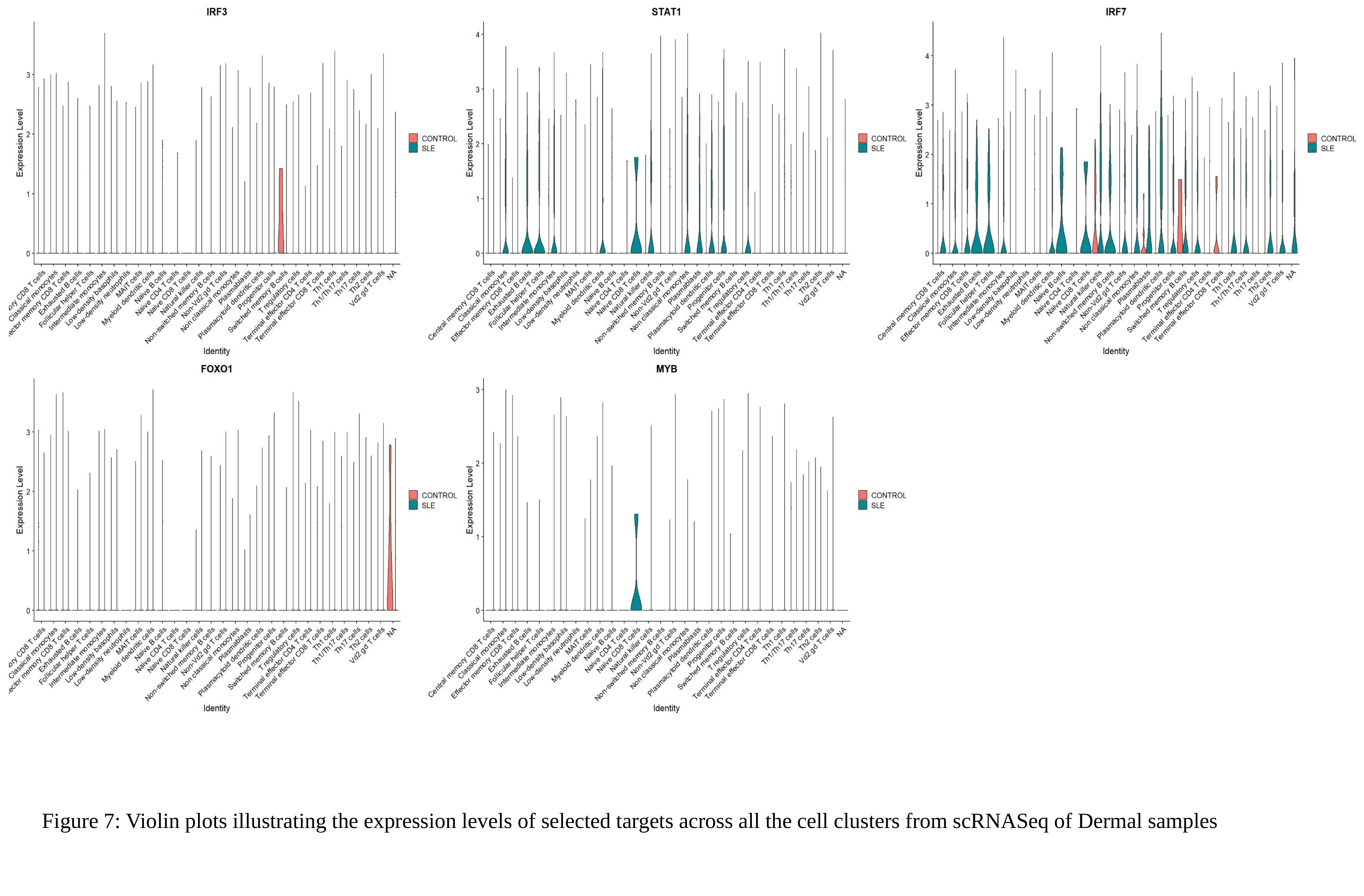

Figure 7: Violin plots illustrating the expression levels of selected targets across all the cell clusters from scRNASeq of Dermal samples

## Slide 8
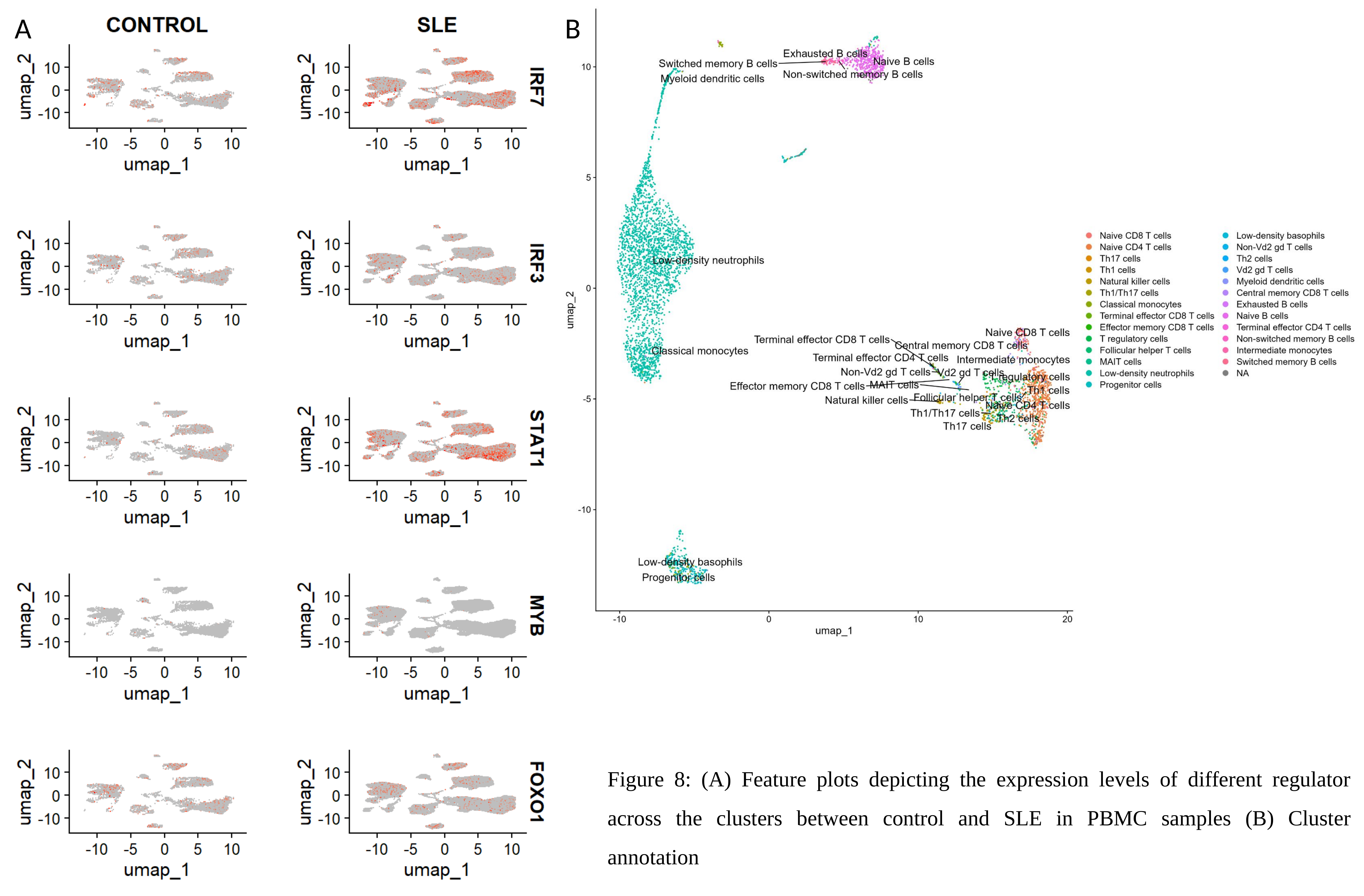

A
B
Figure 8: (A) Feature plots depicting the expression levels of different regulator across the clusters between control and SLE in PBMC samples (B) Cluster annotation

## Slide 9
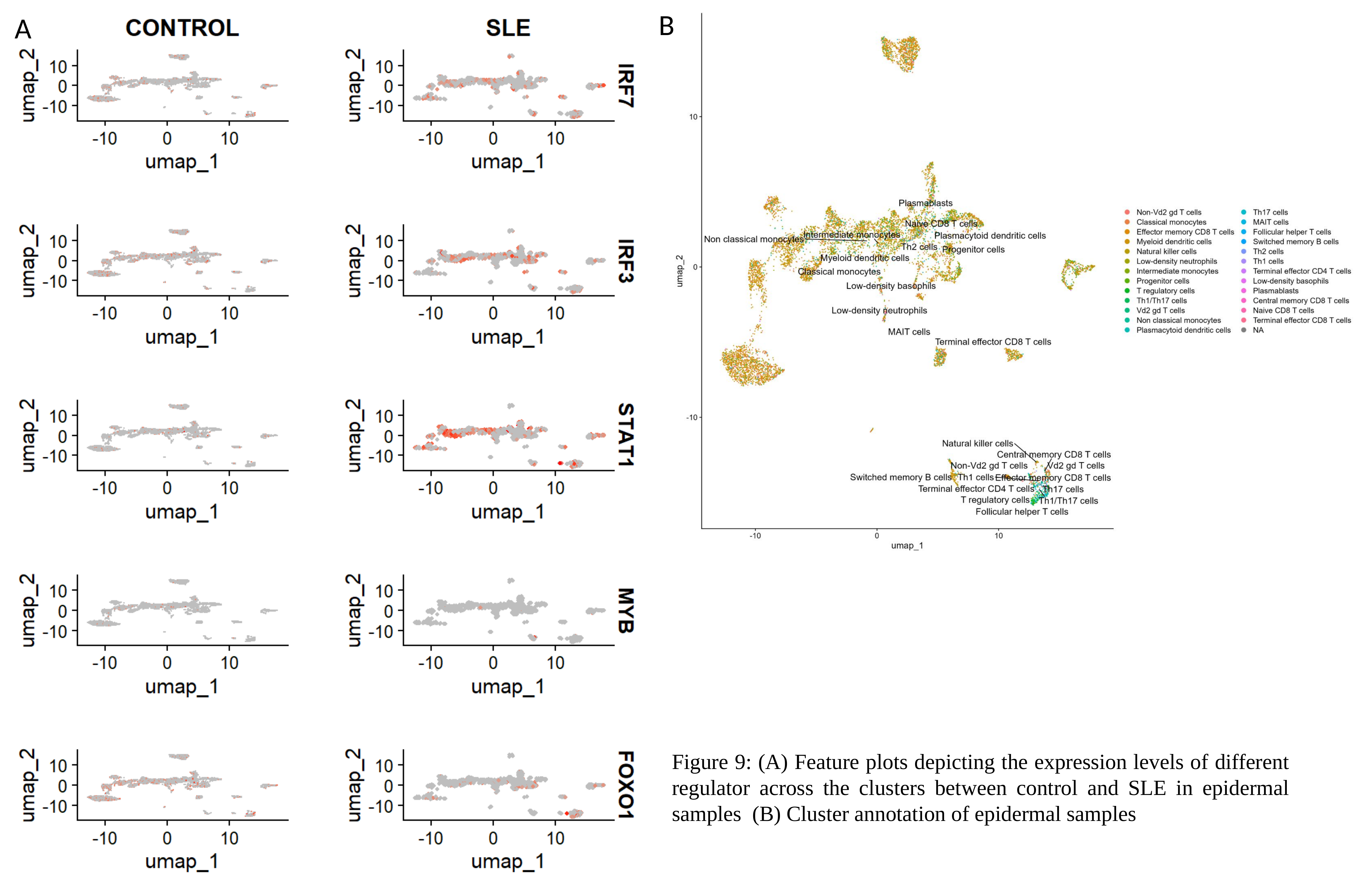

B
A
Figure 9: (A) Feature plots depicting the expression levels of different regulator across the clusters between control and SLE in epidermal samples (B) Cluster annotation of epidermal samples

## Slide 10
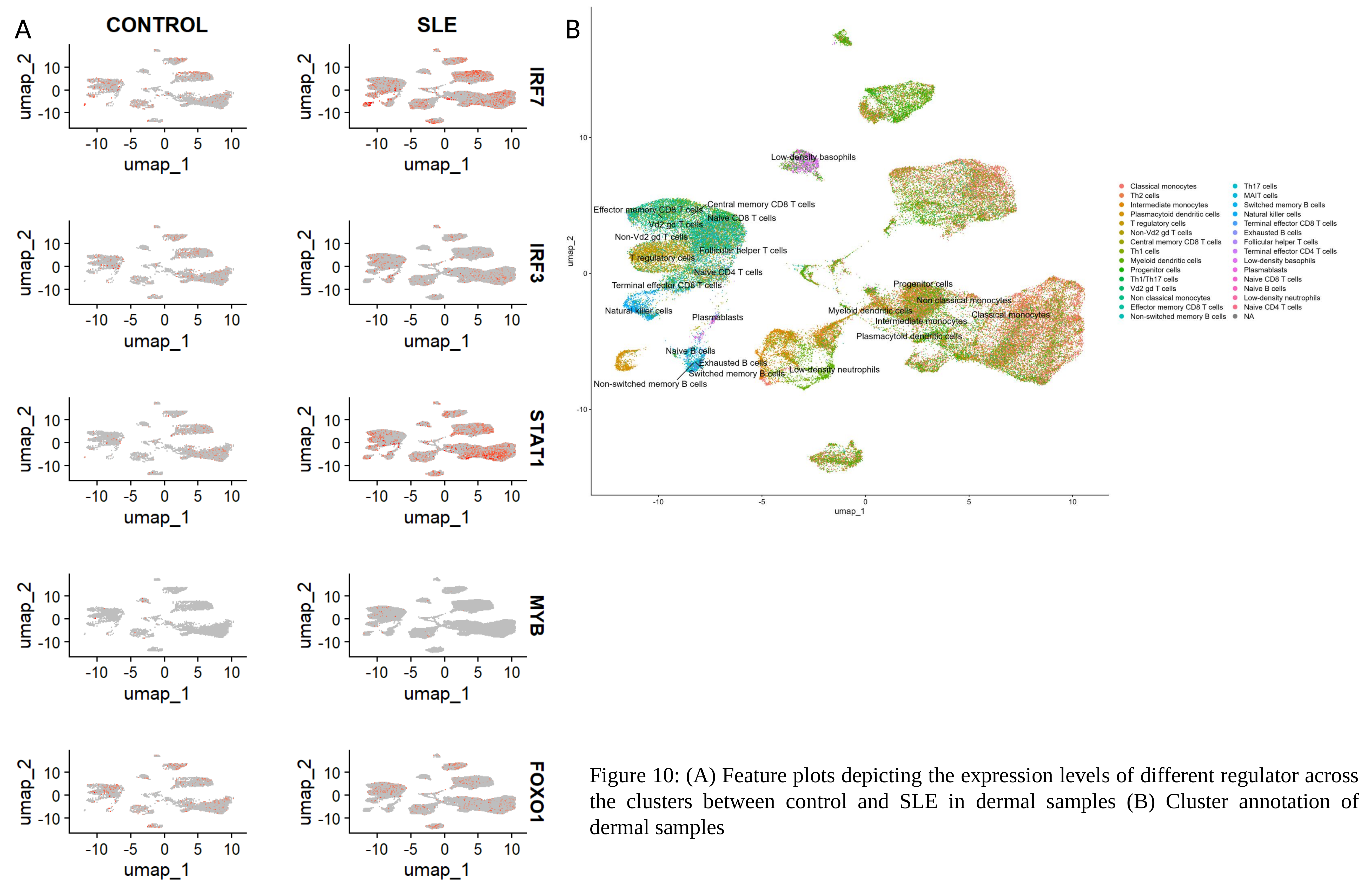

A
B
Figure 10: (A) Feature plots depicting the expression levels of different regulator across the clusters between control and SLE in dermal samples (B) Cluster annotation of dermal samples

## Slide 11
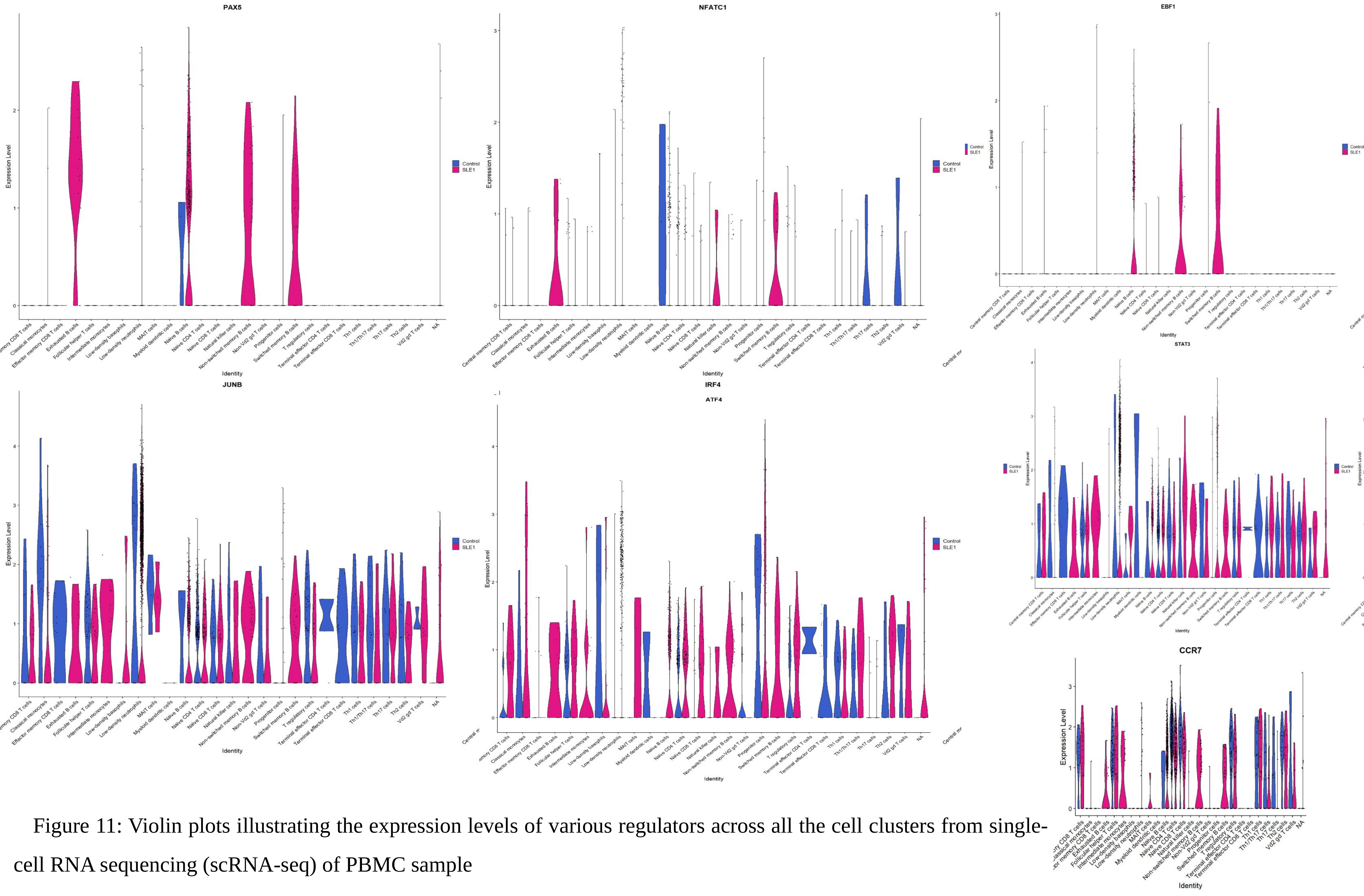

Figure 11: Violin plots illustrating the expression levels of various regulators across all the cell clusters from single-cell RNA sequencing (scRNA-seq) of PBMC sample

## Slide 12
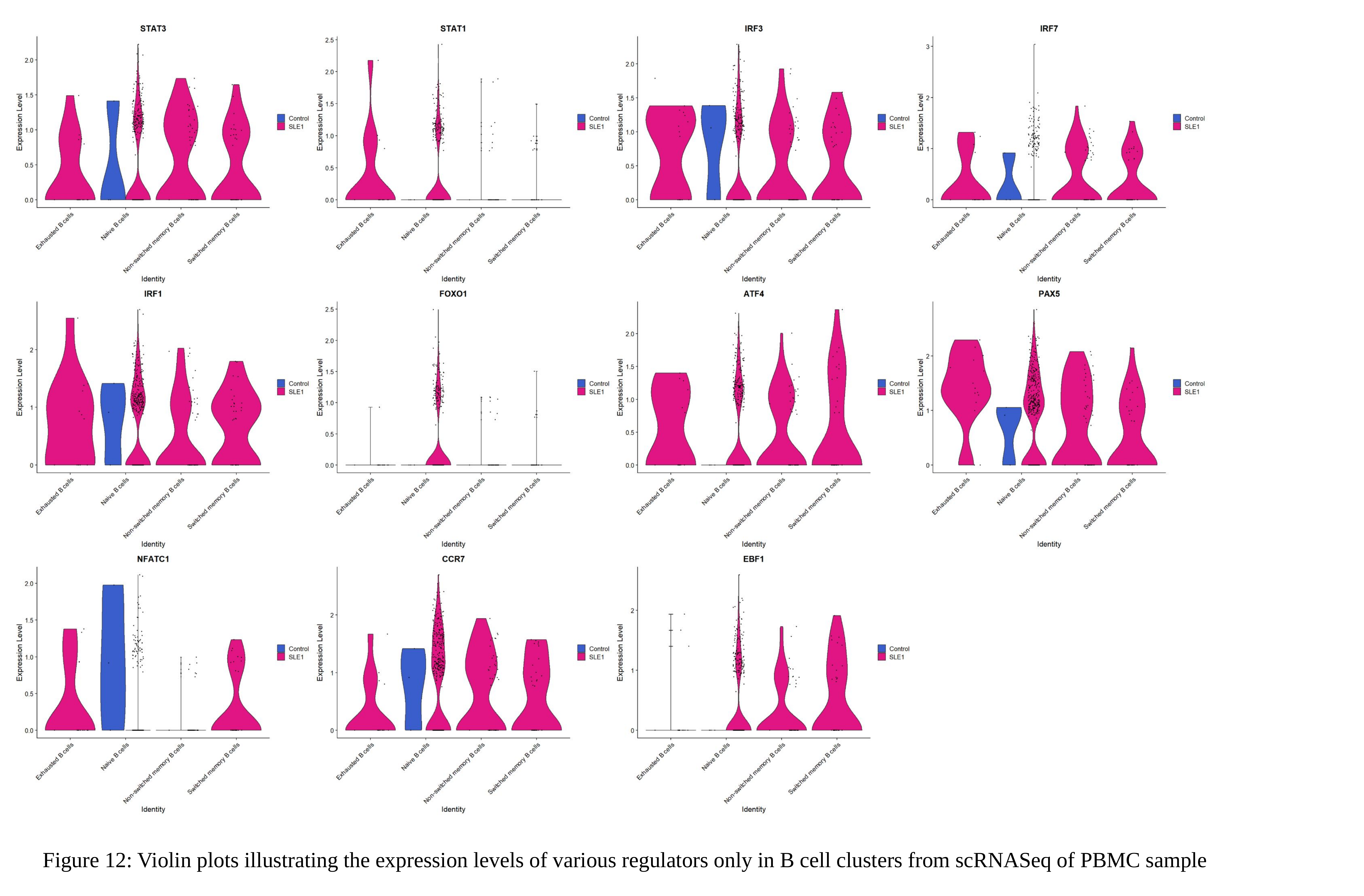

Figure 12: Violin plots illustrating the expression levels of various regulators only in B cell clusters from scRNASeq of PBMC sample

## Slide 13
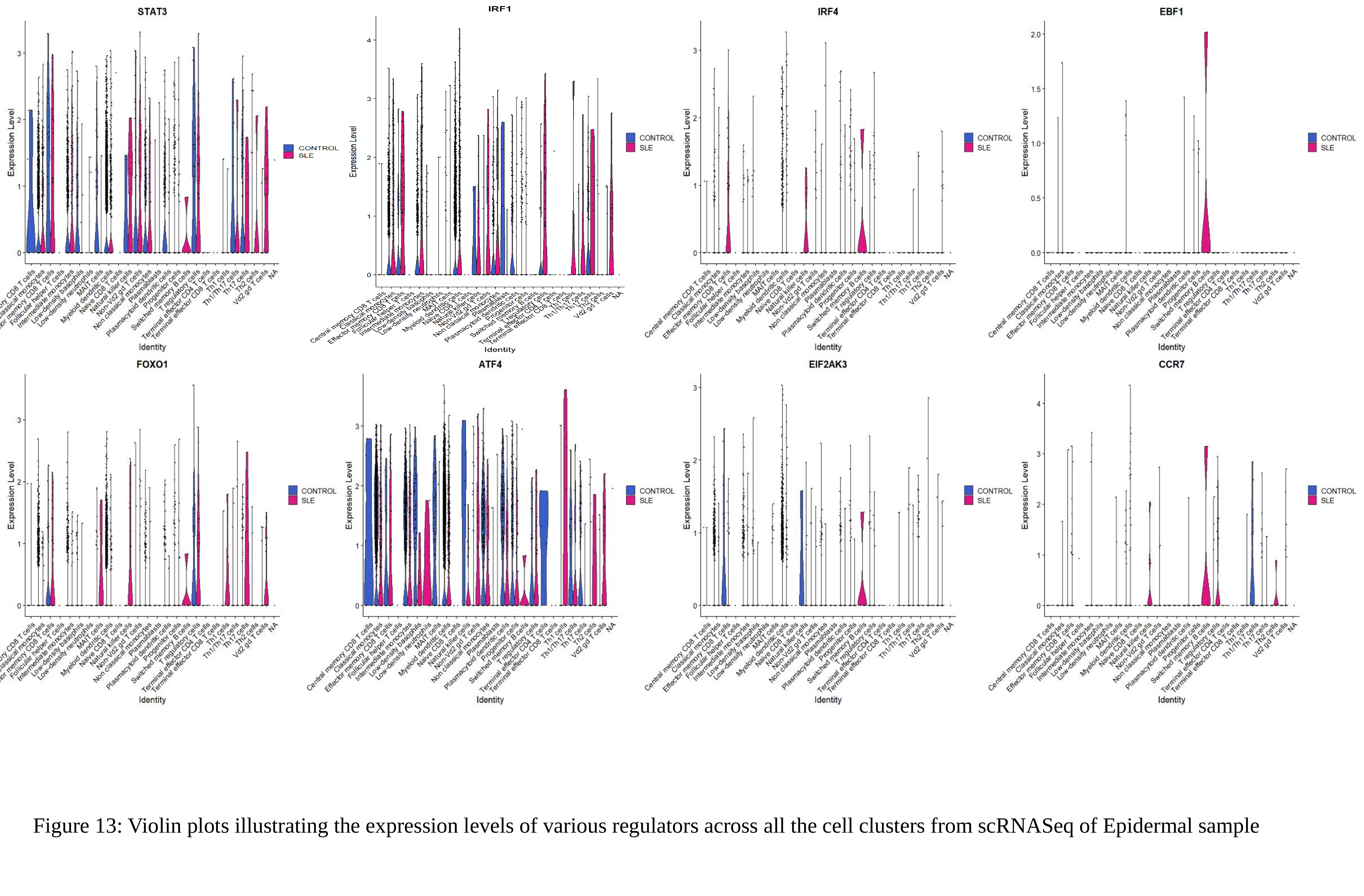

Figure 13: Violin plots illustrating the expression levels of various regulators across all the cell clusters from scRNASeq of Epidermal sample

## Slide 14
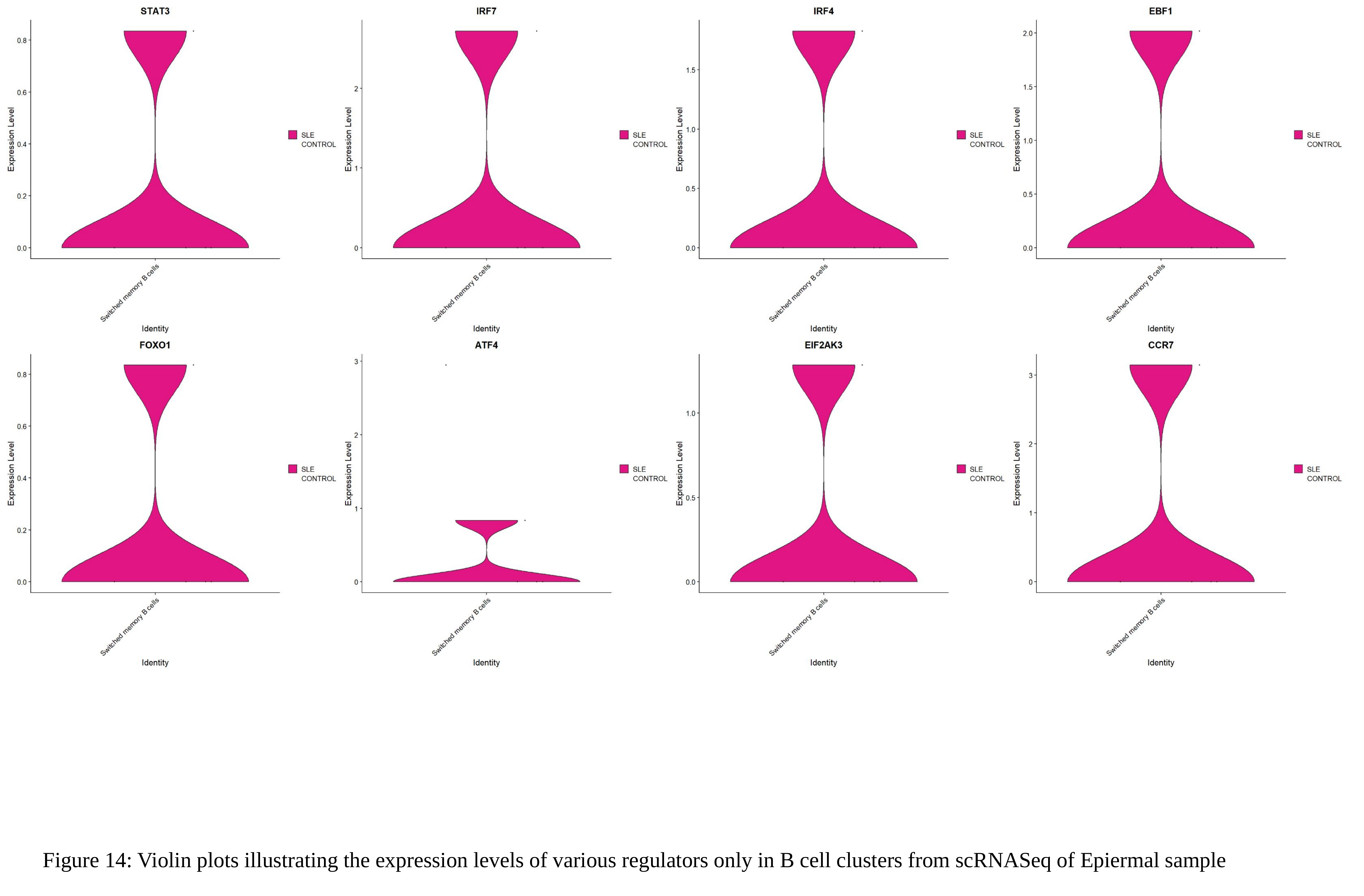

Figure 14: Violin plots illustrating the expression levels of various regulators only in B cell clusters from scRNASeq of Epiermal sample

## Slide 15
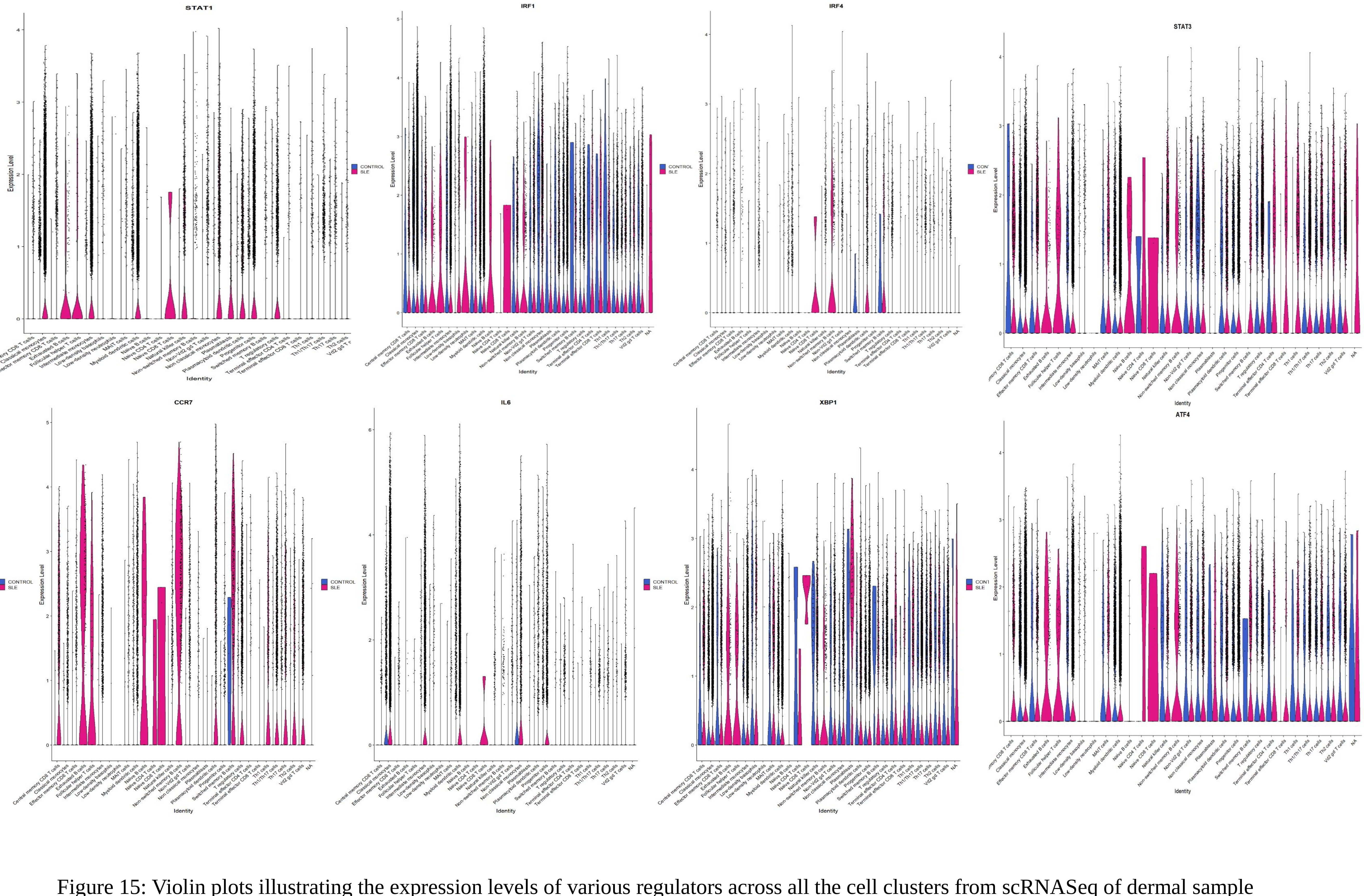

Figure 15: Violin plots illustrating the expression levels of various regulators across all the cell clusters from scRNASeq of dermal sample

## Slide 16
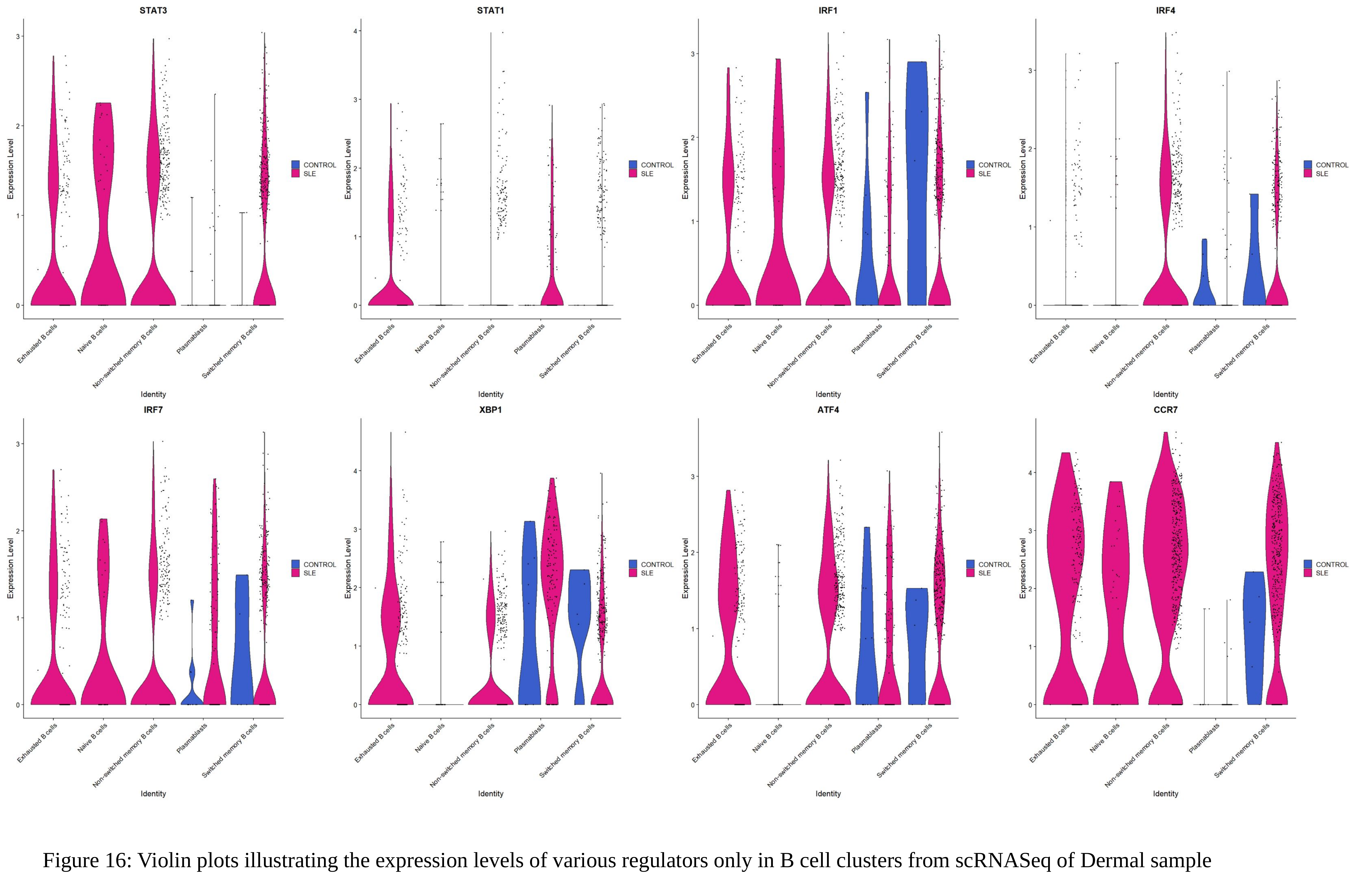

Figure 16: Violin plots illustrating the expression levels of various regulators only in B cell clusters from scRNASeq of Dermal sample
