# Supplementary material for: Mapping integral cell-type-specific interferon-induced gene regulatory networks (GRNs) involved in systemic lupus erythematosus using systems and computational analysis
Source: Heliyon. 2024 Dec 18;11(1):e41342. doi: 10.1016/j.heliyon.2024.e41342 (PMC11751531; doi:10.1016/j.heliyon.2024.e41342)
Supplement: Multimedia component 10 [file mmc10.docx]

- 1. **Gene regulatory circuit for IRF3, IRF7, STAT1, MYB and FOXO1**

Utilizing insights from sections 3.4 and 3.5, combined with comprehensive literature review, we constructed tissue-specific gene regulatory circuits, involving key transcription factors and their target genes **(Figure 5)**.

**NEUTROPHILS**

The neutrophil gene regulatory circuit was deduced from the correlation plot, the regression model, and the literature review. NETosis was considered the endpoint in this gene circuit because one of the important defense responses of neutrophils is the formation of NETs that contain antigens and antimicrobials against microbes. Though it might seem like a defensive benign host process, it's said that its aberrant activation can lead to extensive self-damage by providing a novel source of autoantigens [1], as in SLE [2]. Excessive NET can promote the synthesis of pro-inflammatory cytokine IFN- alpha that can cause direct cytotoxic effects on cells like renal cells. studies reveal that aberrant NETosis can play various roles like autoantibody production, inflammation, and tissue damage at different stages of the disease pathology. Targeting NETosis is seen as an approach to treat SLE [3] [4].

Some important KEGG pathways found using functional enrichment of upregulated genes included Systemic lupus erythematosus, Neutrophil extracellular trap formation, RIG-I-like receptor signaling pathway, and JAK-STAT signaling pathway.

Cell-specific inductive pathways were traced for neutrophils based on the set endpoint and obtained information. The GRN revealed that cell debris that contains self-cytosolic DNA or cytosolic RNA can activate receptors like interferon signaling pathways like cGAS-STING pathway [5] and RIG-I [6] [7]. Furthermore, IFIT3, an upregulated hub gene is proposed to act as a scaffold to promote the interaction of TBK1 and STING, enhancing the activation of IRF3 and IRF7 (upregulated hub gene) to promote the expression of type I IFN [8]. Elevated IFIT3 expression was linked to disease pathology, consistent with a described role for IFIT3 in promoting pathological inflammation in SLE [9]. The interferons activate the JAK/STAT pathway followed by the production of interferon-stimulated genes (ISGs), which most of the hub genes, including ISG15 [10]. ISG-15, a ubiquitin-like protein, is a major component of neutrophil granules and is secreted in response to virus or IFN alpha-beta [11]. Studies also show that neutrophils can be activated by IFN through mitochondrial damage and these activated neutrophils produce reactive oxygens species (ROS) which promotes the release of neutrophil extracellular traps (NETs). Moreover, Protein ISGylation has an inhibitory effect on UPS (ubiquitin–proteasome system), which increases IFN-induced ROS production [12]. Therefore, excess ROS produced during neutrophil activation induces NETosis. Furthermore, increased NET formation and decreased NET degradation enhance the exposure of modified autoantigens that lead to autoantibody production and promote tissue damage [13]. Integrated ncRNA findings revealed that ncRNA LINC00968 is a key regulator for NF-kappaB signaling pathways [14] showed high R2 values with most interferon-stimulated hub genes and ncRNA LINC00487 which is strongly induced by IFNα [15] also showed correlation and high R2 with most hub genes **(Figure 5A)**.

**DENDRITIC CELL**

From the literature review and findings of the regression plots, correlation model, and literature, the GRN was constructed. In dendritic cells (DC), the endpoint was set as antigen presentation, as it is one of the defining characteristics of a dendritic cell, linking innate and adaptive immunity in disease pathology [16] [17]. Though DCs are known to present self-antigens at low levels for immunologic tolerance, once they are activated, they mature and are involved in functions like migration and T-cell priming. Factors like MHC, co-stimulatory molecule expression, and cytokine release affect T-cell priming. Ultimately it results in T cell activation and sustained autoantibody production from B cells. [18] [19] [20] [21].

A few KEGG pathways related to SLE obtained from the functional enrichment of genes are the Cytosolic DNA-sensing pathway, Herpes simplex virus 1 infection, Epstein-Barr virus infection, RIG-I-like receptor signaling pathway, NOD-like receptor signaling pathway, Toll-like receptor signaling pathway, Human papillomavirus infection, NF-kappa B signaling pathway, Apoptosis, Human cytomegalovirus infection, Viral life cycle - HIV-1.

This GRN begins with the stimulation that can be from cytosolic DNA or cytosolic RNA either from a virus such as Epstein-Barr virus (EBV), which can induce type I IFN via the cGAS-STING pathway and viral sensor RIG-I [22], a hub gene that also showed correlation coefficient for ISG above 0.5 respectively. It is also hypothesized that even mitochondrion uncapped RNA can trigger RIG-I pathway [23]. Additionally, OAS is an ISG-encoded antiviral enzyme that processes viral dsRNA into 2¢,5¢-linked oligoadenylate. This unique RNA is subsequently cleaved by RNase L into small products, which potentially activates RIG-I for secondary IFN induction [24]. RIG-I, and MDA5 associate with the adapter IPS-1 via CARD-like domains. IPS-1 is localized to the mitochondrion and initiates signaling leading to the activation of IRF7/3 and NFκB which finally leads to IFN production [25]. TRIM25, a target gene of RIG-1, regulates RIG-I signaling by K63-linked polyubiquitination [26]. The secreted interferons activate the JAK/STAT signaling cascade and lead to the over-production of interferon-stimulated genes (ISGs) [27]. Type I interferons (IFNs), produced by DCs, activate and mature the DCs themselves in a self-reinforcing loop (autocrine manner). This activation includes increased production of IFNs and enhanced expression of molecules like CD80, CD86, and MHC class II on the DC surface. These changes allow DCs to effectively activate T cells. Additionally, type I IFNs directly promote B cell activation, antibody production, and enhance T cell survival and expansion, contributing to a robust immune response [28]. ncRNA LINC01004 which was highly upregulated and showed high correlation for STAT1/2, TRIM25, IRF9, and IRF1, AV plots further showed positive relation to interferon-induced genes, OAS2/3 **(Figure 5B)**.

**T CELLS**

Gene regulatory module (GRM) for T cells was inferred from the correlation plot, regression model, and literature review. The endpoint of GRN of T cell was set as autoimmune induction that can lead to the release of cytokine molecules, activate B cells to produce autoantibodies, and contribute to immune complexes that deposit in tissues and cause inflammation [29] [30][31].

A few of the KEGG pathways of the functional enrichment of T cells include Cytokine-cytokine receptor interaction, Viral protein interaction with cytokine and cytokine receptor, Chemokine signaling pathway, Systemic lupus erythematosus, Epstein-Barr virus infection, JAK-STAT signaling pathway, and Cytosolic DNA-sensing pathway.

CD4+ T cells are activated either by antigen-presenting cells like dendritic cells and macrophages [32] or by antigens that trigger the RIG-I pathway [33] leading to interferon production which in turn activates the JAK/STAT pathway producing interferon-stimulated genes [34]. The ISGs, that are hub genes, namely IFI27, RSAD2, ISG15, MX1, OAS1, IFIT1, and IFIT3 are severely overexpressed leading to immune infiltration and causing organ damage like lupus nephritis [35]. Moreover, MIR223, which is upregulated and shows high correlation for interferons, and is said to promote type I interferon production [36] and also promote the development of pathogenic T cell and autoimmune inflammation [37]. STATs are transcription factors that activate several genes and regulators. One of them is BATF2, an upregulated gene that belongs to the AP-1 superfamily, they are involved in the development and function of CD4+ T cells and it is enhanced by TCR/IFN costimulation along with STATs like STAT2 that can promote interleukin expressions like interleukin-10 (IL-10) [38]. It is also seen to form a complex with IRF1, a regulator of ISGs, inducing inflammation (Roy et al., 2015) (Guler et al., 2015) by producing interferons [40]. The correlation plot (Fig:) shows that LINC00487 has a strong relation between regulators STAT1 and MYB, furthermore, a study shows that it regulates interferon regulation [15]. MYB is a transcription factor that is an upregulated differentially expressed gene. It is involved in the development and maturation of T cells [41] and is also involved in the development of crucial high-affinity development B cells and regulates immunological memory [42]. Reports show that it activates several T cell inflammatory genes like BCL2L14 (upregulated gene) (correlation coefficient above 0.5 and R2 value of 0.5947396), MYC [43], LCK (McCracken et al., 1994), and IL7 (upregulated gene) [45]. BCL2L14, also known as BCL-G, contributes to aberrant cell apoptosis leading to autoantigen spread and inappropriate immune response causing impaired homeostasis [46] LCK regulates the TCR signaling and T-cell development [47], further, IL-7 is also an upregulated DEG, and it functions with TCR, mediating the cell activation and leading to autoimmune inflammation [48]. MYC is also a crucial regulator that directly regulates cell proliferation and controls the cell cycle and metabolic reprogramming [49] it is also the central regulator of TCR signaling and contributes to the hyperactivation of the T cell [50]. ncRNA LINC01260 aslo showed high correlation with MYB, further litersture study shows that it is also regulated by the NF-κB pathway [51]. These genes can turn the cell into an autoreactive and pro-inflammatory cell that enhances the production of autoantibodies by stimulating differentiation, proliferation, and maturation of B cells [52]. Autoantibodies can cause tissue damage leading to organ damage and inflammation **(Figure 5C)**.

**B CELLS**

**Activated naïve B cells**

In activated-naïve B cells GRN, autophagy is considered as the endpoint. Autophagy is the process where the cells undergo degradation during stress conditions and recycle damaged organelles, protein aggregated, and other cytoplasmic contents to maintain homeostasis and survive stress conditions [53]. Interestingly, a recent study suggests that autophagy is not just a consequence of B cell activation, but rather a key mechanism exploited by naïve B cells in SLE. The study revealed that autophagy is specifically activated in SLE naïve B cells, potentially serving as a survival strategy for autoreactive cells. Furthermore, the research demonstrated the essential role of autophagy in plasmablast differentiation. In-vitro inhibition of autophagy significantly impaired the ability of B cells to differentiate into plasmablasts [54]. These findings highlight the multifaceted role of autophagy in B cell function in the context of promoting autoimmunity in SLE [55].

Accumulation of misfolded or unfolded proteins in endoplasmic reticulum (ER), results in ER stress (ERS) response. ERS causes immune system dysregulation leading to disorders like SLE. The ERS activates the ERS pathway sensors namely inositol-requiring enzyme-1 (IRE1), and protein kinase R- (PKR-) like ER kinase (PERK). IRE1 and PERK disassociate from GRP78 and phosphorylate eukaryotic initiation factor 2α (elF2α). Phosphorylated eIF2A enhances the translation of ATF4 [56] and other mRNAs that promote proper protein folding [57]. Also, ATF4 contributes to lupus nephritis-induced kidney autophagy and injury [58]. Furthermore, UV exposure can damage B cells, leading to cell death and loss of the protein ULK1. This, in turn, promotes the activity of STING and the constant production of IFNs. Interferons are also known to induce autophagy by JAK/STAT3, PI3K/AKT/mTOR, and MAPK pathways, enhancing their function in antigen presentation, and a positive feedback loop for the production of interferons [59]. Another pathway that stimulates autophagy is where interleukin-10 activates STAT3 [60], and STAT3 is known to interact with FOXO1 [61]. Further, FOXO1 is a mediator of autophagy [62], and it also activates autophagy-related protein (ATG8) [63]. Interestingly, various studies have shown that the upregulated non-coding RNA LINC00641 plays a role in autophagy [64] [65] [66]. Also, LINC00641 shows a positive correlation and high significance (R2 value = 0.9179705) with FOXO1.

**Antibody-secreting cells**

In ASCs GRN lymph node migration and cell proliferation were considered as the endpoints. Increased expression of CCR7 in ASCs may indicate that they are migrated back to the lymph nodes [67] where they encounter the autoantigens and become more stimulated to produce autoreactive antibodies. Also, studies suggest that CCR7 might act as a survival signal [68] for B cells and this might help the cells resist apoptosis. Furthermore, the uncontrolled proliferation [69] of ASCs could lead to a massive increase in antibody production within tissues [70] (Chen et al, 2023a).

Systemic lupus erythematosus (SLE) antibody-secreting cells (ASCs) exhibit a marked increase in genes associated with ASC differentiation and survival. This includes upregulation of interleukin-10 (IL10), interleukin-6 (IL6), adhesion molecules (ICAM1, CD69), chemokine receptor CXCR4, and B cell receptor (BCR) signaling components JUNB and FOS [71].

B cell receptor (BCR) signaling stimulates the activation of transcription factors, NFATC1, NF-κB, and AP-1. These factors cooperatively regulate the expression of the CCR7 gene [68]. Moreover, along with other regulatory mechanisms FOXO1 is known to regulate the expression of CCR7. FOXO1 is also known to induce B lineage gene expression along with EBF1 [72] (Kerdiles et al, 2009) Also, EBF1 by the activation of AKT pathway [74] contributes to the development, activation, and proliferation of the B cells [75]. Furthermore, upregulated EBF1 enhances B cell function, proliferation [76] and increases the production of co-stimulatory molecules CD40, CD80, and CD86 on the B cell surface [77]. Lastly, to commit cells to B cell fate, PAX5 activated by EBF1 acts at different and multiple levels [78]. Lastly, interferons are key players in SLE that are activated by regulators IRFs and STATs. With regard to ASCs, interferons are known to promote the B cell maturation to ASCs along with IL6 [79]. Research indicates that the upregulated non-coding RNA LINC01619 can activate or increase the expression of FOXO1, which in turn can lead to ASC proliferation [80].

**REFERENCES**

[1] Y. Yu, K. Su, Neutrophil Extracellular Traps and Systemic Lupus Erythematosus, J Clin Cell Immunol 4 (2013). https://doi.org/10.4172/2155-9899.1000139.

[2] R. Salemme, L.N. Peralta, S.H. Meka, N. Pushpanathan, J.J. Alexander, The Role of NETosis in Systemic Lupus Erythematosus, J Cell Immunol 1 (2019) 33. https://doi.org/10.33696/IMMUNOLOGY.1.008.

[3] M. Wang, T. Ishikawa, Y. Lai, D. Nallapothula, R.R. Singh, Diverse Roles of NETosis in the Pathogenesis of Lupus, Front Immunol 13 (2022) 895216. https://doi.org/10.3389/FIMMU.2022.895216/BIBTEX.

[4] N. Hanata, M. Ota, Y. Tsuchida, Y. Nagafuchi, T. Okamura, H. Shoda, K. Fujio, Serum extracellular traps associate with the activation of myeloid cells in SLE patients with the low level of anti-DNA antibodies, Scientific Reports 2022 12:1 12 (2022) 1–14. https://doi.org/10.1038/s41598-022-23076-1.

[5] A. Decout, J.D. Katz, S. Venkatraman, A. Ablasser, The cGAS–STING pathway as a therapeutic target in inflammatory diseases, Nature Reviews Immunology 2021 21:9 21 (2021) 548–569. https://doi.org/10.1038/s41577-021-00524-z.

[6] M. Shrivastav, T.B. Niewold, Nucleic Acid Sensors and Type I Interferon Production in Systemic Lupus Erythematosus, Front Immunol 4 (2013). https://doi.org/10.3389/FIMMU.2013.00319.

[7] J. Rehwinkel, M.U. Gack, RIG-I-like receptors: their regulation and roles in RNA sensing, Nature Reviews Immunology 2020 20:9 20 (2020) 537–551. https://doi.org/10.1038/s41577-020-0288-3.

[8] H. Ye, X. Wang, L. Wang, X. Chu, X. Hu, L. Sun, M. Jiang, H. Wang, Z. Wang, H. Zhao, X. Yang, J. Wang, Full high-throughput sequencing analysis of differences in expression profiles of long noncoding RNAs and their mechanisms of action in systemic lupus erythematosus, Arthritis Res Ther 21 (2019) 1–17. https://doi.org/10.1186/S13075-019-1853-7/FIGURES/7.

[9] H. V. Mears, T.R. Sweeney, Better together: the role of IFIT protein-protein interactions in the antiviral response, J Gen Virol 99 (2018) 1463–1477. https://doi.org/10.1099/JGV.0.001149.

[10] L. Yang, S. Chen, Q. Zhao, C. Pan, L. Peng, Y. Han, L. Li, J. Ruan, J. Xia, H. Yang, F. Xu, G. Cheng, Histone deacetylase 3 contributes to the antiviral innate immunity of macrophages by interacting with FOXK1 to regulate STAT1/2 transcription, Cell Rep 38 (2022) 110302. https://doi.org/10.1016/J.CELREP.2022.110302.

[11] L.M. Bramer, R.D. Hontz, A.J. Eisfeld, A.C. Sims, Y.-M. Kim, K.G. Stratton, C.D. Nicora, M.A. Gritsenko, A.A. Schepmoes, O. Akasaka, M. Koga, T. Tsutsumi, M. Nakamura, I. Nakachi, R. Baba, H. Tateno, S. Suzuki, H. Nakajima, H. Kato, K. Ishida, M. Ishii, Y. Uwamino, K. Mitamura, V.L. Paurus, E.S. Nakayasu, I.K. Attah, A.G. Letizia, K.M. Waters, T.O. Metz, K. Corson, Y. Kawaoka, V.R. Gerbasi, Multi-omics Characterization of Neutrophil Extracellular Trap Formation in Severe and Mild COVID-19 Infections, MedRxiv (2022) 2022.04.26.22274196. https://doi.org/10.1101/2022.04.26.22274196.

[12] J.B. Fan, S. Miyauchi-Ishida, K.I. Arimoto, D. Liu, M. Yan, C.W. Liu, B. Gyorffy, D.E. Zhang, Type I IFN induces protein ISGylation to enhance cytokine expression and augments colonic inflammation, Proc Natl Acad Sci U S A 112 (2015) 14313–14318. https://doi.org/10.1073/PNAS.1505690112/SUPPL_FILE/PNAS.201505690SI.PDF.

[13] Y. Peng, X. Wu, S. Zhang, C. Deng, L. Zhao, M. Wang, Q. Wu, H. Yang, J. Zhou, L. Peng, X. Luo, Y. Chen, A. Wang, Q. Xiao, W. Zhang, Y. Zhao, X. Zeng, Y. Fei, The potential roles of type I interferon activated neutrophils and neutrophil extracellular traps (NETs) in the pathogenesis of primary Sjögren’s syndrome, Arthritis Res Ther 24 (2022). https://doi.org/10.1186/S13075-022-02860-4.

[14] D. Liu, Y. Wan, N. Qu, Q. Fu, C. Liang, L. Zeng, Y. Yang, LncRNA-FAM66C Was Identified as a Key Regulator for Modulating Tumor Microenvironment and Hypoxia-Related Pathways in Glioblastoma, Front Public Health 10 (2022). https://doi.org/10.3389/FPUBH.2022.898270/PDF.

[15] J. Inamo, K. Suzuki, M. Takeshita, Y. Kassai, M. Takiguchi, R. Kurisu, Y. Okuzono, S. Tasaki, S. Tasaki, A. Yoshimura, T. Takeuchi, Identification of novel genes associated with dysregulation of B cells in patients with primary Sjögren’s syndrome, Arthritis Res Ther 22 (2020) 1–11. https://doi.org/10.1186/S13075-020-02248-2/FIGURES/5.

[16] J. Klarquist, Z. Zhou, N. Shen, E.M. Janssen, Dendritic Cells in Systemic Lupus Erythematosus: From Pathogenic Players to Therapeutic Tools, Mediators Inflamm 2016 (2016). https://doi.org/10.1155/2016/5045248.

[17] J. Liu, X. Zhang, X. Cao, Dendritic cells in systemic lupus erythematosus: From pathogenesis to therapeutic applications, J Autoimmun 132 (2022) 102856. https://doi.org/10.1016/J.JAUT.2022.102856.

[18] C. Kaewraemruaen, P. Ritprajak, N. Hirankarn, Allergy and Immunology Dendritic cells as key players in systemic lupus erythematosus, (n.d.). https://doi.org/10.12932/AP-070919-0639.

[19] J.P. Mackern-Oberti, C. Llanos, C.A. Riedel, S.M. Bueno, A.M. Kalergis, Contribution of dendritic cells to the autoimmune pathology of systemic lupus erythematosus, Immunology 146 (2015) 497–507. https://doi.org/10.1111/IMM.12504.

[20] A.K. Hopp, A. Rupp, V. Lukacs-Kornek, Self-antigen presentation by dendritic cells in autoimmunity, Front Immunol 5 (2014) 77232. https://doi.org/10.3389/FIMMU.2014.00055/BIBTEX.

[21] J.H. Fransen, J. V.D. Vlag, J. Ruben, G.J. Adema, J.H. Berden, L.B. Hilbrands, The role of dendritic cells in the pathogenesis of systemic lupus erythematosus, Arthritis Res Ther 12 (2010) 207. https://doi.org/10.1186/AR2966.

[22] J. Pothlichet, T.B. Niewold, D. Vitour, B. Solhonne, M.K. Crow, M. Si-Tahar, A loss-of-function variant of the antiviral molecule MAVS is associated with a subset of systemic lupus patients, EMBO Mol Med 3 (2011) 142. https://doi.org/10.1002/EMMM.201000120.

[23] RIG-I regulation of interferon signature in SLE - Lupus Research, (n.d.). https://www.lupusresearch.org/for-researchers/funded-research/grant/rig-i-regulation-of-interferon-signature-in-sle/ (accessed May 30, 2024).

[24] S.L. Schwartz, G.L. Conn, RNA regulation of the antiviral protein 2’−5’-oligoadenylate synthetase (OAS), Wiley Interdiscip Rev RNA 10 (2019) e1534. https://doi.org/10.1002/WRNA.1534.

[25] X. Hu, S. Goswami, J. Qiu, Q. Chen, S. Laverdure, B.T. Sherman, T. Imamichi, Profiles of Long Non-Coding RNAs and mRNA Expression in Human Macrophages Regulated by Interleukin-27, Int J Mol Sci 20 (2019). https://doi.org/10.3390/IJMS20246207.

[26] M. Martín-Vicente, L.M. Medrano, S. Resino, A. García-Sastre, I. Martínez, TRIM25 in the regulation of the antiviral innate immunity, Front Immunol 8 (2017) 293221. https://doi.org/10.3389/FIMMU.2017.01187/BIBTEX.

[27] H. Kato, S.W. Oh, T. Fujita, RIG-I-Like Receptors and Type I Interferonopathies, J Interferon Cytokine Res 37 (2017) 207–213. https://doi.org/10.1089/JIR.2016.0095.

[28] J. Klarquist, Z. Zhou, N. Shen, E.M. Janssen, Dendritic Cells in Systemic Lupus Erythematosus: From Pathogenic Players to Therapeutic Tools, Mediators Inflamm 2016 (2016). https://doi.org/10.1155/2016/5045248.

[29] A. Suárez-Fueyo, S.J. Bradley, G.C. Tsokos, T cells in Systemic Lupus Erythematosus, Curr Opin Immunol 43 (2016) 32. https://doi.org/10.1016/J.COI.2016.09.001.

[30] V.R. Moulton, G.C. Tsokos, Abnormalities of T cell signaling in systemic lupus erythematosus, Arthritis Res Ther 13 (2011) 1–10. https://doi.org/10.1186/AR3251/FIGURES/3.

[31] R.W. Hoffman, T cells in the pathogenesis of systemic lupus erythematosus, Clinical Immunology 113 (2004) 4–13. https://doi.org/10.1016/J.CLIM.2004.05.001.

[32] S.J. Gaudino, P. Kumar, Cross-Talk Between Antigen Presenting Cells and T Cells Impacts Intestinal Homeostasis, Bacterial Infections, and Tumorigenesis, Front Immunol 10 (2019) 360. https://doi.org/10.3389/FIMMU.2019.00360.

[33] M. Kandasamy, A. Suryawanshi, S. Tundup, J.T. Perez, M. Schmolke, S. Manicassamy, B. Manicassamy, RIG-I Signaling Is Critical for Efficient Polyfunctional T Cell Responses during Influenza Virus Infection, PLoS Pathog 12 (2016). https://doi.org/10.1371/JOURNAL.PPAT.1005754.

[34] M.G. Katze, Y. He, M. Gale, Viruses and interferon: a fight for supremacy, Nature Reviews Immunology 2002 2:9 2 (2002) 675–687. https://doi.org/10.1038/nri888.

[35] X. Zhao, L. Zhang, J. Wang, M. Zhang, Z. Song, B. Ni, Y. You, Identification of key biomarkers and immune infiltration in systemic lupus erythematosus by integrated bioinformatics analysis, J Transl Med 19 (2021). https://doi.org/10.1186/S12967-020-02698-X.

[36] L. Chen, Y. Song, L. He, X. Wan, L. Lai, F. Dai, Y. Liu, Q. Wang, MicroRNA-223 Promotes Type I Interferon Production in Antiviral Innate Immunity by Targeting Forkhead Box Protein O3 (FOXO3), J Biol Chem 291 (2016) 14706. https://doi.org/10.1074/JBC.M115.700252.

[37] T. Satoorian, B. Li, X. Tang, J. Xiao, W. Xing, W. Shi, K.H.W. Lau, D.J. Baylink, X. Qin, MicroRNA223 promotes pathogenic T-cell development and autoimmune inflammation in central nervous system in mice, Immunology 148 (2016) 326–338. https://doi.org/10.1111/IMM.12611.

[38] U. Govender, B. Corre, Y. Bourdache, S. Pellegrini, F. Michel, Type I interferon-enhanced IL-10 expression in human CD4 T cells is regulated by STAT3, STAT2, and BATF transcription factors, J Leukoc Biol 101 (2017) 1181–1190. https://doi.org/10.1189/JLB.2A0416-187RR.

[39] S. Roy, R. Guler, S.P. Parihar, S. Schmeier, B. Kaczkowski, H. Nishimura, J.W. Shin, Y. Negishi, M. Ozturk, R. Hurdayal, A. Kubosaki, Y. Kimura, M.J.L. de Hoon, Y. Hayashizaki, F. Brombacher, H. Suzuki, Batf2/Irf1 induces inflammatory responses in classically activated macrophages, lipopolysaccharides, and mycobacterial infection, J Immunol 194 (2015) 6035–6044. https://doi.org/10.4049/JIMMUNOL.1402521.

[40] BATF2 Contributes to Interferon Dysregulation in SLE Keratinocytes - ACR Meeting Abstracts, (n.d.). https://acrabstracts.org/abstract/batf2-contributes-to-interferon-dysregulation-in-sle-keratinocytes/ (accessed April 2, 2024).

[41] Y.K. Lieu, A. Kumar, A.G. Pajerowski, T.J. Rogers, E.P. Reddy, Requirement of c-myb in T cell development and in mature T cell function, Proc Natl Acad Sci U S A 101 (2004) 14853–14858. https://doi.org/10.1073/PNAS.0405338101/ASSET/64A6E77F-ACF4-44FC-B206-0BA3AD089DD5/ASSETS/GRAPHIC/ZPQ0420462170006.JPEG.

[42] T. Bender, c-Myb in CD4 T cells is crucial for recall antibody responses, (n.d.). https://grantome.com/grant/NIH/R56-AI108767-01 (accessed April 1, 2024).

[43] J.P. Cogswell, P.C. Cogswell, W.M. Kuehl, A.M. Cuddihy, T.M. Bender, U. Engelke, K.B. Marcu, J.P.-Y. Ting, Mechanism of c-myc regulation by c-Myb in different cell lineages, Mol Cell Biol 13 (1993) 2858–2869. https://doi.org/10.1128/MCB.13.5.2858-2869.1993.

[44] S. McCracken, S. Leung, R. Bosselut, J. Ghysdael, N.G. Miyamoto, Myb and Ets related transcription factors are required for activity of the human lck type I promoter, Oncogene 9 (1994) 3609–3615. https://pubmed.ncbi.nlm.nih.gov/7970721/ (accessed April 1, 2024).

[45] J.Z. Qin, C.L. Zhang, J. Kamarashev, R. Dummer, G. Burg, U. Döbbeling, Interleukin-7 and interleukin-15 regulate the expression of the bcl-2 and c-myb genes in cutaneous T-cell lymphoma cells, Blood 98 (2001) 2778–2783. https://doi.org/10.1182/BLOOD.V98.9.2778.

[46] M.L. Hartman, M. Czyz, BCL-G: 20 years of research on a non-typical protein from the BCL-2 family, Cell Death & Differentiation 2023 30:6 30 (2023) 1437–1446. https://doi.org/10.1038/s41418-023-01158-5.

[47] U. Bommhardt, B. Schraven, L. Simeoni, Beyond TCR Signaling: Emerging Functions of Lck in Cancer and Immunotherapy, Int J Mol Sci 20 (2019). https://doi.org/10.3390/IJMS20143500.

[48] B.R. Lawson, R. Gonzalez-Quintial, T. Eleftheriadis, M.A. Farrar, S.D. Miller, K. Sauer, D.B. McGavern, D.H. Kono, R. Baccala, A.N. Theofilopoulos, Interleukin-7 is required for CD4(+) T cell activation and autoimmune neuroinflammation, Clin Immunol 161 (2015) 260–269. https://doi.org/10.1016/J.CLIM.2015.08.007.

[49] R. Wang, C.P. Dillon, L.Z. Shi, S. Milasta, R. Carter, D. Finkelstein, L.L. McCormick, P. Fitzgerald, H. Chi, J. Munger, D.R. Green, The transcription factor Myc controls metabolic reprogramming upon T lymphocyte activation, Immunity 35 (2011) 871–882. https://doi.org/10.1016/J.IMMUNI.2011.09.021.

[50] D.J. Perry, A.A. Titov, E.S. Sobel, T.M. Brusko, L. Morel, Immunophenotyping Reveals Distinct Subgroups of Lupus Patients Based on their Activated T Cell Subsets, Clin Immunol 221 (2020) 108602. https://doi.org/10.1016/J.CLIM.2020.108602.

[51] D.M. Wu, X.R. Han, X. Wen, S. Wang, Y.J. Wang, M. Shen, S.H. Fan, J. Zhuang, Z.F. Zhang, Q. Shan, M.Q. Li, B. Hu, C.H. Sun, J. Lu, Y.L. Zheng, Long Non-Coding RNA LINC01260 Inhibits the Proliferation, Migration and Invasion of Spinal Cord Glioma Cells by Targeting CARD11 Via the NF-κB Signaling Pathway, Cell Physiol Biochem 48 (2018) 1563–1578. https://doi.org/10.1159/000492279.

[52] Z. Li, Z. Wang, T. Sun, S. Liu, S. Ding, L. Sun, Identifying key genes in CD4+ T cells of systemic lupus erythematosus by integrated bioinformatics analysis, Front Genet 13 (2022) 941221. https://doi.org/10.3389/FGENE.2022.941221/BIBTEX.

[53] Y. Wang, Z.H. Qin, Coordination of autophagy with other cellular activities, Acta Pharmacologica Sinica 2013 34:5 34 (2013) 585–594. https://doi.org/10.1038/aps.2012.194.

[54] A.J. Clarke, U. Ellinghaus, A. Cortini, A. Stranks, A.K. Simon, M. Botto, T.J. Vyse, T.K. Kvien, Autophagy is activated in systemic lupus erythematosus and required for plasmablast development, Ann Rheum Dis 74 (2015) 912–920. https://doi.org/10.1136/ANNRHEUMDIS-2013-204343.

[55] Y. yuan Qi, X. jie Zhou, H. Zhang, Autophagy and immunological aberrations in systemic lupus erythematosus, Eur J Immunol 49 (2019) 523–533. https://doi.org/10.1002/EJI.201847679.

[56] M. Luhr, M.L. Torgersen, P. Szalai, A. Hashim, A. Brech, J. Staerk, N. Engedal, The kinase PERK and the transcription factor ATF4 play distinct and essential roles in autophagy resulting from tunicamycin-induced ER stress, J Biol Chem 294 (2019) 8197–8217. https://doi.org/10.1074/JBC.RA118.002829.

[57] H.Y. Li, L.F. Huang, X.R. Huang, D. Wu, X.C. Chen, J.X. Tang, N. An, H.F. Liu, C. Yang, Endoplasmic Reticulum Stress in Systemic Lupus Erythematosus and Lupus Nephritis: Potential Therapeutic Target, J Immunol Res 2023 (2023). https://doi.org/10.1155/2023/7625817.

[58] J. Jin, L. Zhao, W. Zou, W. Shen, H. Zhang, Q. He, Activation of Cyclooxygenase-2 by ATF4 During Endoplasmic Reticulum Stress Regulates Kidney Podocyte Autophagy Induced by Lupus Nephritis, Cell Physiol Biochem 48 (2018) 753–764. https://doi.org/10.1159/000491904.

[59] X. Liu, H. Qin, J. Xu, The role of autophagy in the pathogenesis of systemic lupus erythematosus, Int Immunopharmacol 40 (2016) 351–361. https://doi.org/10.1016/J.INTIMP.2016.09.017.

[60] A.P. Hutchins, D. Diez, D. Miranda-Saavedra, The IL-10/STAT3-mediated anti-inflammatory response: recent developments and future challenges, Brief Funct Genomics 12 (2013) 489. https://doi.org/10.1093/BFGP/ELT028.

[61] H.M. Oh, C.R. Yu, I. Dambuza, B. Marrero, C.E. Egwuagu, STAT3 Protein Interacts with Class O Forkhead Transcription Factors in the Cytoplasm and Regulates Nuclear/Cytoplasmic Localization of FoxO1 and FoxO3a Proteins in CD4+ T Cells, J Biol Chem 287 (2012) 30436. https://doi.org/10.1074/JBC.M112.359661.

[62] Y. Zhao, J. Yang, W. Liao, X. Liu, H. Zhang, S. Wang, D. Wang, J. Feng, L. Yu, W.G. Zhu, Cytosolic FoxO1 is essential for the induction of autophagy and tumour suppressor activity, Nat Cell Biol 12 (2010) 665–675. https://doi.org/10.1038/NCB2069.

[63] T. Shpilka, H. Weidberg, S. Pietrokovski, Z. Elazar, Atg8: an autophagy-related ubiquitin-like protein family, Genome Biol 12 (2011) 226. https://doi.org/10.1186/GB-2011-12-7-226.

[64] X.B. Wang, H. Wang, H.Q. Long, D.Y. Li, X. Zheng, LINC00641 regulates autophagy and intervertebral disc degeneration by acting as a competitive endogenous RNA of miR-153-3p under nutrition deprivation stress, J Cell Physiol 234 (2019) 7115–7127. https://doi.org/10.1002/JCP.27466.

[65] D. Meng, S. Zhao, L. Wu, X. Ma, D. Zhao, Z. Li, LINC00641 impeded the malignant biological behaviors of papillary thyroid carcinoma cells via interacting with IGF2BP1 to reduce GLI1 mRNA stability, Hum Exp Toxicol 42 (2023). https://doi.org/10.1177/09603271231180856/ASSET/IMAGES/LARGE/10.1177_09603271231180856-FIG7.JPEG.

[66] Y. Hu, Y. Su, X. Lei, H. Zhao, L. Wang, T. Xu, J. Guo, W. Yang, X. Zhang, LINC00641/miR-582-5p mediate oxaliplatin resistance by activating autophagy in gastric adenocarcinoma, Scientific Reports 2020 10:1 10 (2020) 1–11. https://doi.org/10.1038/s41598-020-70913-2.

[67] C. Beauvillain, P. Cunin, A. Doni, M. Scotet, S. Jaillon, M.L. Loiry, G. Magistrelli, K. Masternak, A. Chevailler, Y. Delneste, P. Jeannin, CCR7 is involved in the migration of neutrophils to lymph nodes, Blood 117 (2011) 1196–1204. https://doi.org/10.1182/BLOOD-2009-11-254490.

[68] C. Cuesta-Mateos, F. Terrón, M. Herling, CCR7 in Blood Cancers – Review of Its Pathophysiological Roles and the Potential as a Therapeutic Target, Front Oncol 11 (2021). https://doi.org/10.3389/FONC.2021.736758.

[69] L. Li, D. Zhang, X. Cao, EBF1, PAX5, and MYC: regulation on B cell development and association with hematologic neoplasms, Front Immunol 15 (2024) 1320689. https://doi.org/10.3389/FIMMU.2024.1320689/BIBTEX.

[70] B. Dema, N. Charles, Autoantibodies in SLE: Specificities, Isotypes and Receptors, Antibodies 5 (2016). https://doi.org/10.3390/ANTIB5010002.

[71] W. Chen, S.-H. Hong, S.A. Jenks, F.A. Anam, C.M. Tipton, M.C. Woodruff, J.R. Hom, K.S. Cashman, C.E. Faliti, X. Wang, S. Kyu, C. Wei, C.D. Scharer, T. Mi, S. Hicks, L. Hartson, D.C. Nguyen, A. Khosroshahi, S. Lee, Y. Wang, R. Bugrovsky, Y. Ishii, F.E.-H. Lee, I. Sanz, SLE Antibody-Secreting Cells Are Characterized by Enhanced Peripheral Maturation and Survival Programs, Res Sq (2023). https://doi.org/10.21203/RS.3.RS-3016327/V1.

[72] J.Y. Jang, I. Hwang, H. Pan, J. Yao, L. Alinari, E. Imada, C. Zanettini, M.J. Kluk, Y. Wang, Y. Lee, H. V. Lin, X. Huang, M. Di Liberto, Z. Chen, K. V. Ballman, L.C. Cantley, L. Marchionni, G. Inghirami, O. Elemento, R.A. Baiocchi, S. Chen-Kiang, S. Belvedere, H. Zheng, J. Paik, A FOXO1-dependent transcription network is a targetable vulnerability of mantle cell lymphomas, J Clin Invest 132 (2022). https://doi.org/10.1172/JCI160767.

[73] Y.M. Kerdiles, D.R. Beisner, R. Tinoco, A.S. Dejean, D.H. Castrillon, R.A. DePinho, S.M. Hedrick, Foxo1 links homing and survival of naive T cells by regulating L-selectin, CCR7 and interleukin 7 receptor, Nat Immunol 10 (2009) 176–184. https://doi.org/10.1038/NI.1689.

[74] M.J. Griffin, Y. Zhou, S. Kang, X. Zhang, T.S. Mikkelsen, E.D. Rosen, Early B-cell Factor-1 (EBF1) Is a Key Regulator of Metabolic and Inflammatory Signaling Pathways in Mature Adipocytes, J Biol Chem 288 (2013) 35925. https://doi.org/10.1074/JBC.M113.491936.

[75] I. Györy, S. Boller, R. Nechanitzky, E. Mandel, S. Pott, E. Liu, R. Grosschedl, Transcription factor Ebf1 regulates differentiation stage-specific signaling, proliferation, and survival of B cells, Genes Dev 26 (2012) 668. https://doi.org/10.1101/GAD.187328.112.

[76] Y. Zhou, T. Luo, R. Xue, Q. Liu, L. Hui, EBF1 Promotes Cell Proliferation, Migration and Inhibits Cell Apoptosis in AML, Blood 140 (2022) 11486–11486. https://doi.org/10.1182/BLOOD-2022-159318.

[77] S. Luo, Y. Liu, G. Liang, M. Hao, H. Wu, Y. Liang, X. Qiu, Y. Tan, Y. Dai, S. Yung, T.M. Chan, Q. Lu, The role of microRNA-1246 in the regulation of B cell activation and the pathogenesis of systemic lupus erythematosus, Clin Epigenetics 7 (2015). https://doi.org/10.1186/S13148-015-0063-7.

[78] C. Murre, “Big bang” of B-cell development revealed, Genes Dev 32 (2018) 93–95. https://doi.org/10.1101/GAD.311357.118.

[79] G. Jego, A.K. Palucka, J.P. Blanck, C. Chalouni, V. Pascual, J. Banchereau, Plasmacytoid dendritic cells induce plasma cell differentiation through type I interferon and interleukin 6, Immunity 19 (2003) 225–234. https://doi.org/10.1016/S1074-7613(03)00208-5.

[80] X. Bai, J. Geng, X. Li, J. Wan, J. Liu, Z. Zhou, X. Liu, Long Noncoding RNA LINC01619 Regulates MicroRNA-27a/Forkhead Box Protein O1 and Endoplasmic Reticulum Stress-Mediated Podocyte Injury in Diabetic Nephropathy, Https://Home.Liebertpub.Com/Ars 29 (2018) 355–376. https://doi.org/10.1089/ARS.2017.7278.
